# Symmetry-restoring quantum phase transition in a two-dimensional spinor condensate

**DOI:** 10.1038/s41598-018-30876-x

**Published:** 2018-08-20

**Authors:** A. L. Chudnovskiy, V. Cheianov

**Affiliations:** 1 1. Institut für Theoretische Physik, Universität Hamburg, Jungiusstr 9, D-20355 Hamburg, Germany; 2 Instituut-Lorentz, Universiteit Leiden, P.O. Box 9506, 2300 RA Leiden, The Netherlands

## Abstract

Bose Einstein condensates of spin-1 atoms are known to exist in two different phases, both having spontaneously broken spin-rotation symmetry, a ferromagnetic and a polar condensate. Here we show that in two spatial dimensions it is possible to achieve a quantum phase transition from a polar condensate into a singlet phase symmetric under rotations in spin space. This can be done by using particle density as a tuning parameter. Starting from the polar phase at high density the system can be tuned into a strong-coupling intermediate-density point where the phase transition into a symmetric phase takes place. By further reducing the particle density the symmetric phase can be continuously deformed into a Bose-Einstein condensate of singlet atomic pairs. We calculate the region of the parameter space where such a molecular phase is stable against collapse.

## Introduction

Spinor atomic quantum gases provide a platform for experimental realization of a wide variety of quantum phases with exotic properties. The high degree of control of inter-atomic interactions opens access to quantum orders that have no analogs in solid state systems^1–12^. Exotic properties of a ground state of a spinor Bose-Einstein condensate (BEC) can generally be related to the arrangement of the internal degrees of freedom of the constituent atoms. The simplest nontrivial example is provided by a BEC of spin-1 bosons described by the Hamiltonian

1 $$H=\int \,d{\bf{x}}[\frac{{\hslash }^{2}}{2m}\nabla {\psi }_{a}^{\dagger }\nabla {\psi }_{a}+\frac{{c}_{0}}{2}{\psi }_{a}^{\dagger }{\psi }_{b}^{\dagger }{\psi }_{b}{\psi }_{a}+\frac{{c}_{2}}{2}{\psi }_{a}^{\dagger }{\psi }_{a^{\prime} }^{\dagger }{\psi }_{b^{\prime} }{\psi }_{b}{{\bf{F}}}_{ab}\cdot {{\bf{F}}}_{a^{\prime} b^{\prime} }].$$

Here the spin-index *a* assumes the values 1, 0, −1, and the matrix-valued vector **F** consists of the *S* = 1 representation of *SU*(2) group generators. The interaction constants *c*_0_ and *c*_2_ describe the scalar and spin interactions respectively. Since the interaction in Eq. (1) is spin-conserving, it is convenient to represent the interaction term according to the spin-0 and spin-2 scattering channels (the s-wave scattering in the spin-1 channel is suppressed by the symmetry of the bosonic wave function). Thereby the effective interaction constants become *g*_0_ = *c*_0_ − 2*c*_2_, and *g*_2_ = *c*_0_ + *c*_2_. Mean field analysis of the Hamiltonian (1) reveals two stable zero-temperature phases^13,14^: the ferromagnetic BEC condensate for *c*_2_ < 0, and the polar condensate for anti-ferromagnetic interactions, *c*_2_ > 0. In both phases, the *SU*(2) symmetry is spontaneously broken, resulting in divergent magnetic susceptibilities at the transition, and gapless spin-waves as elementary low-energy excitations. Detailed analysis of different symmetry breaking patterns and associated soft modes in terms of the nonlinear *σ*-model in given in ref.^15^.

Curiously enough, although there seem to be no fundamental reasons precluding the existence of the *SU*(2)-symmetric Bose-condensate, such a singlet phase having no gapless spin-waves is not present on the mean field phase diagram. A theoretical possibility that a singlet phase can emerge as a result of quantum dynamics of the broken symmetry condensate’s zero modes was considered in ref.^16^. However, the *SU*(2) symmetric vacuum constructed in ref.^16^ is unstable against an infinitesimal magnetic field in the thermodynamic limit^17^, signifying a spontaneously broken symmetry^18^. Therefore, the mechanism discussed in ref.^16^ does not lead to the formation of an *SU*(2)-symmetric phase. The idea of an *SU*(2) symmetric Bose-condensate of bound singlet two-particle states in three dimensions was put forward in the work^19^, Bose condensate of singlet pairs in an exactly solvable limit of one-dimensional system was considered in ref.^20^.

In this paper we show that a quantum phase transition from the polar condensate to an *SU*(2)-symmetric phase can be achieved in two dimensions by varying the density of atoms. Our conclusion is based on the interpolation between two limiting cases, each admitting for controllable analytical treatment. The limit of high particle density is amenable to the mean field approach of ref.^13^, where it was found that the polar condensate is the stable ground state for *c*_2_ > 0. In the opposite limit of extremely low particle density, and when *g*_0_ = *c*_0_ − 2*c*_2_ < 0, we demonstrate, that the ground state is a condensate of weakly interacting molecules, each being a bound state of two atoms in a spin-singlet state. Such a singlet molecular condensate (SMC) does not break the *SU*(2) symmetry, and its spin-excitations have a spectral gap equal to the binding energy of a molecule. Such a symmetric phase was earlier described in three dimensions in ref.^19^, and in one dimension in ref.^20^.

In contrast to the metastable quasi-bound molecular states, appearing close to Feshbach resonance, as discussed in ref.^21^, we find the SMC phase thermodynamically stable against both the collapse towards larger atomic clusters and the formation of the polar condensate due to the breakup of molecular pairs. With increasing density, the molecules lose their individuality and the system becomes analytically intractable, in analogy with the BEC to BCS crossover in fermionic quantum gases^22,23^. However, unlike fermion systems, the *SU*(2) symmetry persists only up to a critical density, at which the symmetry breaking phase transition to the polar condensate takes place. This density roughly corresponds to the inter-atomic distance comparable with the size of a single molecule. We would like to stress that we are only considering the zero temperature case. At finite temperatures the situation is more complex due to the effects of long range thermal fluctuations^24,25^.

## Results

### Bound singlet pairs

We begin our analysis with recalling some peculiarities of the Hamiltonian Eq. (1) in two dimensions. The strength of the two-body interactions is determined by two coupling constants $m{g}_{\nu }/{\hslash }^{2}$ (*ν* = 0, 2), which are dimensionless. This implies that at the classical level the Hamiltonian does not have an intrinsic length scale, and hence the symmetry of the mean field ground state is insensitive to the particle density. Beyond the mean field, perturbative quantum corrections to the two-particle scattering amplitudes experience ultraviolet logarithmic divergences, amenable to the renormalization group treatment^26–28^. Thus, in the quantum picture, the parameters *g*_0_ and *g*_2_ should be considered as renormalized coupling constants, which depend on the running energy scale *E* as^26–29^

2 $${g}_{\nu }(E)=\frac{4\pi {\hslash }^{2}}{m}\frac{1}{\mathrm{ln}\,({E}_{\nu }/E)},$$

where *E*_*ν*_ are determined by the ultraviolet (UV) physics. In Eq. (2) it is assumed that the running energy *E* is less than the UV energy cutoff Λ. In the mean field theory, the energy scale *E* is set by the chemical potential, which in the case of the polar condensate is given by *μ* = *c*_0_*n*, where *n* is the particle density^13^. Therefore, on the quantum level, the coupling constants in the Hamiltonian Eq. (1) depend on the particle density.

The mean field treatment of the Hamiltonian Eq. (1) is justified for small coupling constants. In the case of weak repulsive interaction, Λ ≪ *E*_*i*_, the coupling constants decrease with decreasing density. In contrast, any weak attractive coupling (Λ ≫ *E*_*i*_, *g*_*i*_ < 0) increases with decreasing energy, driving the Hamiltonian into a strong coupling regime. Thus, while at high densities the Hamiltonian can still be analyzed within the mean field theory approach, in the low density limit the mean field theory breaks down. As is often the case, in such a limit the fundamental degrees of freedom can no longer be used for a meaningful description of the system’s properties. Rather one has to work with the weakly interacting physical degrees of freedom, if those can be identified. In order to understand what those are in the present case, we first consider dilution so extreme, that only two atoms are present in the system. Since any attractive two particle interaction in two dimensions creates a bound state^30^, the attraction in the singlet channel, *g*_0_ < 0, binds the two atoms into a spin-singlet molecule. Such a molecule is characterized by its binding energy *E*_0_, and its size $d=\sqrt{m{E}_{0}/\hslash }$. At small but finite particle density, $n\ll \hslash /(m{E}_{0})$, such molecules form a weakly nonideal Bose gas, in which intermolecular collisions occur only rarely. The effect of such collisions depends on the sign of the intermolecular interaction. For repulsive interaction, a stable *SU*(2) symmetric Bose-Einstein condensate of molecules is formed. Such a condensate is described by a scalar Gross-Pitaevskii functional with a running interaction constant *c*_*M*_(*E*) > 0, and with the UV cutoff determined by the binding energy of a molecule. In contrast, intermolecular attraction, *c*_*M*_ < 0, leads to the instability of the system against collapse.

Focusing on the *c*_*M*_ > 0 case, the energy of the molecular condensate is given by *E*_*M*_ = *N*(−*E*_0_ + *c*_*M*_*n*/4)/2, where *N* and *n* denote the total number and the density of atoms respectively. In order for the molecular pair condensate to describe the thermodynamic equilibrium, *E*_*M*_ needs to be less than the energy of the polar condensate given by *E*_*p*_ = *c*_0_*Nn*/2. Obviously, this criterion is fulfilled for low atomic density. With increasing particle density, however, the interactions between molecules get increasingly more complex due to the molecules probing each other’s internal structure in collision events. Such effects must result in *nd*^2^ corrections to the ground state energy, making the regime of intermediate densities *nd*^2^ ~ 1 analytically intractable. It is in this regime that the singlet phase becomes thermodynamically unstable and the transition into the polar phase eventually occurs. For a condensate constrained to two dimensions by a parabolic potential of oscillator length ${\ell }_{0}$ we estimate the critical density as

3 $${n}_{c} \sim {d}^{-2}=\frac{1}{{\ell }_{0}^{2}}{e}^{\pi {\ell }_{0}/{a}_{3{\rm{D}}}^{\mathrm{(0)}}},$$

where ${a}_{3{\rm{D}}}^{\mathrm{(0)}}$ is the scattering length characterizing the collision of two atoms in the *F* = 0 scattering channel in three dimensions.

### Intermolecular interaction

An important question to be addressed is whether the regime of *c*_*M*_ > 0 can be accessed in principle despite the presence of an attractive interaction which binds two atoms into a pair. In order to investigate this issue we perform a detailed quantitative analysis of the intermolecular interaction, employing the Skorniakov and Ter-Martirosian (STM) formalism as described in the methodology section. We find that in the situation of a competition between the attraction in the *F* = 0 and repulsion in the *F* = 2 scattering channels, the sign of the intermolecular interaction constant is determined by the value of a single parameter

4 $$\lambda =\frac{{\ell }_{0}}{\sqrt{2\pi }}(\frac{1}{|{a}_{{\rm{3D}}}^{\mathrm{(0)}}|}+\frac{1}{|{a}_{{\rm{3D}}}^{\mathrm{(2)}}|}).$$

where ${a}_{3{\rm{D}}}^{(F)}$ denotes the scattering length characterizing the collision of two atoms in the *F* = 0, 2 scattering channel in three dimensions. Smaller values of *λ* correspond to greater repulsion in the *F* = 2 channel (see Eq. (4)) and less attraction between the molecules. At *λ* < *λ*_*c*_ = (1.4 ± 0.1) the overall interaction between the molecules is found repulsive. Therefore, for *λ* < *λ*_*c*_ the condensate of singlet pairs is stabilized against further collapse. Eq. (4) allows to reformulate the condition *λ* < 1.4 in terms of the three-dimensional scattering lengths and the oscillator length of confinement potential

5 $$\frac{{\ell }_{0}}{|{a}_{{\rm{3D}}}^{\mathrm{(2)}}|} < 1.4\sqrt{2\pi }-\frac{{\ell }_{0}}{|{a}_{{\rm{3D}}}^{\mathrm{(0)}}|}.$$

It is worth noting, that similar analysis of the collision process between an atom and a singlet molecule rules out the existence of a three-particle bound state in the whole range of parameter *λ* (see Supplementary Information for detailed calculations).

## Discussion

In this paper we predict the existence of *SU*(2) symmetric ground state of spin-1 Bose gas in two dimensions. Being a superfluid of singlet pair of atoms (singlet molecules), this state breaks the *SU*(2) × *U*(1) symmetry of the systems to *SU*(2), in contrast to a complete breaking of both *SU*(2) and *U*(1) symmetries pertinent to the previously known polar condensate^13^. The emergence of a stable *SU*(2) symmetric ground state is due to an unconventional mechanism of restoration of a spontaneously broken symmetry in quantum field theory. In contrast to the restoration of symmetry by long-range fluctuations of the order parameter field, in the present case the symmetry is restored by the formation of bound states of elementary particles (atoms), which happens at a finite length scale. Precursors of this mechanism can be seen in the renormalization flow of coupling constants describing the Gross- Pitaevskii Hamiltonian of the system. In the case of attraction in the *F* = 0 channel renormalization flow drives the theory into a strong couping regime at low particle density, which signals the formation of bound singlet states of two atoms. We expect the existence of a quantum phase transition between the low-density *SU*(2) symmetric condensate of singlet molecules and the polar one-particle condensate at high densities, where the mean field treatment of the system is appropriate. The phase transition point lies in the strong coupling regime, no matter whether the theory is formulated in terms of atomic or molecular degrees of freedom. Therefore, although we reliably predict the two ground states with different symmetries, our approach neither predicts the exact position of the phase transition point nor gives any insight as to the transition kind. One possible route to the analysis of the nature of the phase transition could be through the modification of the mean field description of the phase transition in atom-molecular systems employed in refs^9–12^ in the vicinity of the Feshbach resonance. It is worth noting that logarithmic renormalization of coupling constants is a general feature of two-dimensional non-relativistic systems, hence we expect the mechanism of the formations of the bound state be of general importance for two dimensional cold atomic gases.

Next we discuss the experimental conditions for the realization of the SMC phase. Assuming a typical oscillator length of the parabolic confinement ${\ell }_{0}\sim 200$ Å^31–34^, and the scattering length $|{a}_{3{\rm{D}}}^{(0)}|=100$ Å, Eq. (3) gives the density *n*_*c*_ ~ 10^9^ cm^−2^, which means that for a typical densities $n \sim {10}^{8}{{\rm{cm}}}^{-2}$ the condensate of singlet molecules should form. Increasing temperature of the singlet condensate, we expect the Kosterlitz-Thouless transition from the BEC to a gas of singlet pairs at ${T}_{c} \sim \frac{\pi {\hslash }^{2}n}{4m}$, where *n* is the density of molecules, and *m* is a mass of the atom. For a density *n* = 10^−8^ cm^−2^ we obtain in the case of ^41^K *T*_*c*_ ~ 4nK. Eq. (5) specifies the conditions for the three-dimensional scattering length in *S* = 0 and *S* = 2 channels, and the characteristic length of the confinement potential, at which the two-dimensional condensate of singlet pairs is stable against collapse. For example, for the oscillator length ${\ell }_{0}\sim 200$ Å, we get the condition $|{a}_{3{\rm{D}}}^{(2)}|,|{a}_{3{\rm{D}}}^{(0)}|\gtrsim 100$ Å. Experimentally, the regime specified by Eq. (5) can be realized with help of optical Feshbach resonance^35–37^ or using the radio-frequency dressed atomic states^38^. Another interesting realization of the BEC of singlet pairs can be acheaved in the ^85^Rb–^87^Rb mixtures, where the ^85^Rb - ^87^Rb molecule with total angular momentum 1 plays the role of a spin-1 boson^11,12^, and the singlet pairs are created by two ^85^Rb - ^87^Rb molecules. In this case the spin is realized as an orbital degree of freedom of neutral spinless atoms, therefore the scattering length can be tuned using the magnetic Feshbach resonance while preserving the *SU*(2) spin symmetry.

Analysis of stability of SMC represents an example of stabilization of BEC built of composite objects (bound singlet pairs) by competition between the attractive and repulsive interactions in different scattering channels. Technical analysis of stability of SMC can prove useful for a broad range of fields, where the existence and stability of molecular BEC remains a long-standing problem, such as BEC of para-hydrogen molecules^39,40^, or exciton-polaritons^41,42^.

## Methods

### Skorniakov and Ter-Martirosian formalism in two dimensions

Here we recall the Skorniakov and Ter-Martirosian formalism, see refs^43,44^, focusing on the peculiarities of the two-dimensional case. This formalism is effective for determining the scattering amplitudes and the energies of the bound states in few-body scattering problems with short range scattering potentials. The STM approach is based on the observation that in a system with a short range two-body interaction potential, many-particle dynamics is fully determined by the two-particle scattering amplitudes in the s-wave channel. The latter in turn can be emulated with the Bethe-Peierls (BP) boundary condition imposed on the many-body wave function

6 $${\frac{1}{\psi }\frac{\partial \psi }{\partial {r}_{ij}}|}_{{r}_{ij}={R}_{0}}=-\,\frac{1}{a}.$$

Here *r*_*ij*_ is the distance between the colliding particles *i* and *j*. The parameter *R*_0_ is related to the UV energy cutoff ${\rm{\Lambda }}={\hslash }^{2}/(m{R}_{0}^{2})$. The scattering amplitude at energy *E* only depends on the dimensionless ratios *a*/*R*_0_ and *E*/Λ, which reflects the renormalization group symmetry of the system in two dimensions. The set of points satisfying *r*_*ij*_ = *R*_0_ defines a hyper-cylinder in the 2*N* dimensional configuration space. The union of such hyper-cylinders for all pairs *i*, *j* is called the scattering surface, which we denote by ${\mathscr{S}}$. The formal solution of the *N*-particle scattering problem can be written in the form^44^

7 $${\psi }_{E}({\bf{r}})={\psi }_{E}^{0}({\bf{r}})+{\int }_{{\mathscr{S}}}\,d{\bf{r}}^{\prime} {G}_{E}({\bf{r}}-{\bf{r}}{\boldsymbol{^{\prime} }}){f}_{E}({\bf{r}}{\boldsymbol{^{\prime} }}\mathrm{)}.$$

Here *ψ*_*E*_(**r**) denotes the exact many-particle wave functions at a given energy, ${\psi }_{E}^{0}({\bf{r}})$ denotes the incoming wave, *G*_*E*_(**r** − **r**′) denotes the Green function, describing propagation of particles without collisions, and *f*_*E*_(**r**′) is an auxiliary function defined on the scattering surface. In the STM formalism one applies the BP boundary condition Eq. (6) to Eq. (7), which results in a closed integral equation for the function *f*_*E*_(**r**′). The latter is also called the STM equation.

In the case of two particles, the function *f*_*E*_(**r**) has no spatial dependence, and the explicit solution of the STM equation for *E* > 0 reads (see Supplementary Information for a detailed derivation)

8 $${f}_{E}=\frac{1}{2\mu {R}_{0}[\mathrm{ln}\,(\sqrt{2\mu E}{R}_{0}{e}^{a/{R}_{0}})-i\pi \mathrm{/2}]},$$

where *μ* denotes the reduced mass. Analytical continuation of the solution Eq. (8) to the upper half-plane (the unphysical sheet) of complex energy results in a pole at the negative energy axis at

9 $$E=-\,{E}_{0}=-\,\frac{1}{2\mu {R}_{0}^{2}}{e}^{-2a/{R}_{0}}.$$

This pole directly translates into a pole on the unphysical sheet of the scattering amplitude, indicating the existence of a bound bound state at energy −*E*_0_^45^. This correspondence between the singularity of the scattering amplitude and the existence of the bound state can be understood as follows: At the energy −*E*_0_, Eq. (7) has a formal solution without the incoming wave. We note that for the scattering parameter *a* > 0, the energy *E*_0_ is much less than the high-energy cutoff Λ.

It is instructive to relate the effective parameters *a* and *R*_0_ to the microscopic parameters of the trapped Bose condensate. The two-body scattering problem for a parabolically confined two-dimensional system was explicitely solved in refs^29,46^. In this system, the UV length scale is set by the oscillator length of the parabolic confinement potential ${\ell }_{0}$. Comparing the results of^29,46^ with the scattering amplitude obtained from the Bethe-Peierls boundary condition Eq. (6), and setting for convenience the UV length cutoff ${R}_{0}=1.31{\ell }_{0}$, we find

10 $$\frac{a}{{R}_{0}}=-\sqrt{\frac{\pi }{2}}\frac{{\ell }_{0}}{{a}_{{\rm{3D}}}},$$

where *a*_3D_ is the 3-dimensional s-wave scattering length. For the attractive interaction in the singlet channel, ${a}_{{\rm{3D}}}^{\mathrm{(0)}} < 0$ in Eq. (10), we deduce that there is a bound state of two atoms, which is a singlet pair with the binding energy *E*_0_, as given by Eq. (9).

### Derivation of STM-equations for two scattering channels

Next we proceed to the calculation of the effective interaction between two singlet molecules. To this end, we solve the two-pair scattering problem. Since we consider only the scattering events without the dissociation of the pairs, the final state still consists of the two singlet molecules. Moreover, the total spin of the intermediate four-particle state equals 0. Guided by that reason, we introduce the basis of two-pair states in the spin space as follows ${{\rm{\Phi }}}_{i}={|i,4\rangle }_{s}\otimes {|j,k\rangle }_{s}$, where *i* ≠ *j* ≠ *k* ≠ 4. Each state consists of two singlet pairs of atoms, and it is marked by the number of the atom that forms a singlet with the atom 4. The general two-pair wave function reads

11 $${\boldsymbol{\Psi }}({{\bf{r}}}_{1},\,{{\bf{r}}}_{2},\,{{\bf{r}}}_{3},\,{{\bf{r}}}_{4})=\sum _{i\mathrm{=1}}^{3}\,{\chi }_{i}({{\bf{r}}}_{1},\,{{\bf{r}}}_{2},\,{{\bf{r}}}_{3},\,{{\bf{r}}}_{4}){{\rm{\Phi }}}_{i}.$$

Here *χ*_*i*_ describes the spatial part of the wave function and Φ_*i*_ relates to the spin part. Furthermore, we separate the center of mass coordinate of the four atoms, and introduce the following set of Jacobi-coordinates to describe their relative position

12 $${\bf{z}}=({{\bf{r}}}_{3}-{{\bf{r}}}_{4}),\,{\bf{y}}=({{\bf{r}}}_{1}-{{\bf{r}}}_{2}),\,x=\frac{1}{\sqrt{2}}[({{\bf{r}}}_{3}+{{\bf{r}}}_{4})-({{\bf{r}}}_{1}+{{\bf{r}}}_{2}\mathrm{)]}.$$

Since we have two scattering channels (spin 0 and spin 2), there are two sets of BP boundary conditions that read

13 $${{\partial }_{{r}_{ij}}{\hat{P}}_{ij}^{(\nu )}{\boldsymbol{\Psi }}|}_{{r}_{ij}={R}_{0}}={-\frac{1}{{a}_{\nu }}{\hat{P}}_{ij}^{(\nu )}{\boldsymbol{\Psi }}|}_{{r}_{ij}={R}_{0}}.$$

Here *r*_*ij*_ = |**r**_*i*_ − **r**_*j*_|, ${\hat{P}}_{ij}^{(\nu )}$ denotes the projection operator on the subspace, in which the atoms *i*, *j* have the total spin *ν*, (*ν* = 0, 2). Applying Eq. (13) to Eq. (7), we obtain the set of equations for functions ${f}_{ij}^{\nu }$, each having its domain on the hyper-cylinder *r*_*ij*_ = *R*_0_. The symmetry of the bosonic wave function under the permutations of atoms further reduces the number of independent unknown functions to two, *f*_0_(**x**, **z**) and *f*_2_(**x**, **z**), which are related to the scattering amplitudes in the *F* = 0 and *F* = 2 channels respectively. The variable **y** has been eliminated by BP boundary conditions applied on the hyper-cylinder |**r**_1_ − **r**_2_| = |*y*| = *R*_0_. After the Fourier transform in the variables **x** and **z**, the STM equations for the two-channel problem take the form (a detailed derivation is provided in the Supplementary Information)

14 $$\begin{array}{c}{\alpha }_{\nu }({\bf{k}},\,{\bf{p}}){f}_{\nu }({\bf{k}},\,{\bf{p}})=\frac{2\sqrt{\pi }\mathrm{(2}-\varepsilon ){\delta }_{\nu \mathrm{,0}}}{1+{p}^{2}\mathrm{(2}-\varepsilon )}\delta ({\bf{k}}-{\bf{K}})+\int \,\frac{{d}^{2}{\bf{Q}}}{{\mathrm{(2}\pi )}^{2}}\frac{2}{1+{k}^{2}+{p}^{2}+{Q}^{2}}\\ \,\,\,\,\,\,\,\{{f}_{\nu }(\,-\,{\bf{k}},\,{\bf{Q}})+2\sum _{\mu =\mathrm{0,2}}{K}_{\nu \mu }\sum _{s=\pm }{f}_{\mu }(\frac{{\bf{p}}+{\bf{Q}}}{\sqrt{2}},\,s\frac{{\bf{Q}}-({\bf{k}}+{\bf{p}})}{\sqrt{2}})\}.\end{array}$$

Here *ε* = (*E* + 2*E*_0_)/*E*_0_ is the dimensionless energy relative to the rest energy of two isolated molecules. The energy *E*_0_ is given by Eq. (9) for the singlet scattering channel, *a* = *a*_0_. The Fourier conjugates of **x** and **z**, which we denote as **k** and **p** respectively, are measured in units of $\sqrt{|E|}$. The functions *α*_*ν*_(**k**, **p**) are defined by

15 $${\alpha }_{\nu }({\bf{k}},\,{\bf{p}})=\frac{1}{2\pi }\,\mathrm{ln}\,\mathrm{[(2}-\varepsilon \mathrm{)(1}+{k}^{2}+{p}^{2})]-\lambda {\delta }_{\nu \mathrm{,2}}.$$

The scattering in the spin-2 scattering channel enters Eq. (14) through the parameter *λ*, which is defined as

16 $$\lambda =\frac{({a}_{0}+|{a}_{2}|)}{\pi {R}_{0}}=\frac{{\ell }_{0}}{\sqrt{2\pi }}(\frac{1}{|{a}_{{\rm{3D}}}^{\mathrm{(0)}}|}+\frac{1}{|{a}_{{\rm{3D}}}^{\mathrm{(2)}}|}),$$

where the second equation is obtained in virtue of Eq. (10), and we consider the case ${a}_{{\rm{3D}}}^{\mathrm{(0)}} < 0$, corresponding to the attraction in the singlet channel. The first term on the RHS of Eq. (14) represents the incoming wave with the relative momentum of the two molecules **K**. We note, that the incoming wave is only present in the singlet channel, *ν* = 0. The explicit form of the matrix *κ*_*νμ*_ follows from the symmetry of the wave function by spin rotations and permutations of atoms, it reads

17 $$\kappa =(\begin{array}{cc}\mathrm{1/3} & \mathrm{5/9}\\ 1 & \mathrm{1/6}\end{array}).$$

**Figure 1:**
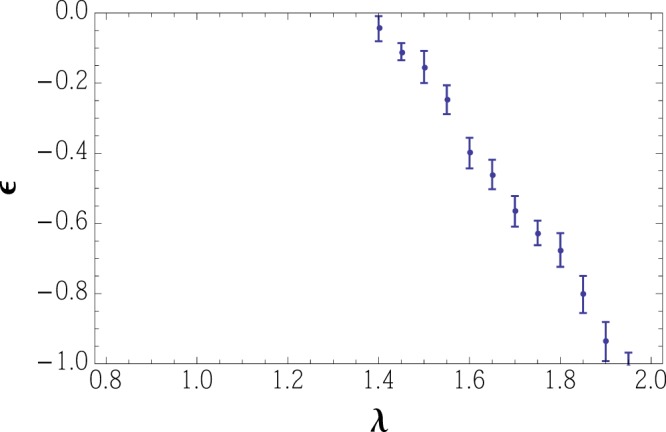
Energy *ε* of the stationary state of Markovian evolution, corresponding to the bound state of two molecules with energy −2 + *ε* (in units of *E*_0_) as a function of parameter *λ* measuring the relative strength of attraction in spin-0 and repulsion in spin-2 channels (see Eq. (4)), averaged over 20 independent runs of Markovian evolution. The two-molecule bound state appears for *λ *≈ 1.4 ± 0.1 as a solution with negative *ε*, which determines the boundary for the stability of the SMC ground state.

If the interaction between two molecules is attractive, then a two-molecule bound complex is formed in two dimensions, which mathematically is indicated as a nontrivial solution of STM equations without the incoming wave. It may also happen that the combination of attraction in the *F* = 0 channel and repulsion in the *F* = 2 channel results in the overall repulsion between two singlet pairs. In that case no four-particle bound state should exist. We solve STM equations for the scattering of two singlet molecules numerically, using the method of stochastic Markovian evolution with branching^47^, looking for a four-particle bound state with energy −2*E*_0_ + *ε*, *ε* < 0, i. e. below the energy of two non-interacting molecules (for details see Sec. 0.3). Our numerical results are shown in Fig. 1. Decreasing *λ*, which corresponds to increase of repulsive interactions in the spin-2 channel (see Eq. (4)) reduces the absolute value of *ε*, at which a bound state is found, and below *λ*_*c*_ = (1.4 ± 0.1) no bound state exists. Therefore, for *λ* < *λ*_*c*_ the condensate of singlet pairs is stabilized against further collapse.

### Markovian evolution

In this section we describe the mapping of Eq. (14) without incoming wave term on the Markovian evolution process. Let us introduce functions *g*_*ν*_(**k**) defined as

18 $${g}_{\nu }({\bf{k}},\,{\bf{p}})={f}_{\nu }({\bf{k}},\,{\bf{p}})\frac{\mathrm{ln}\,\mathrm{[(2}-\varepsilon \mathrm{)(1}+{k}^{2}+{p}^{2})]}{1+{k}^{2}+{p}^{2}}.$$

After the transformation Eq. (18), equations for *g*_*ν*_(**k**) are written in the form that allows their iterative solution

19 $${g}_{\nu ,n+1}({\bf{k}},\,{\bf{p}})=\sum _{\mu =\mathrm{0,2}}\,\int {d}^{2}{\bf{k}}\text{'}{d}^{2}{\bf{p}}\text{'}{P}_{\nu \mu }({\bf{k}},\,{\bf{p}};\,{\bf{k}}\text{'},\,{\bf{p}}\text{'}){g}_{\nu ,n}({\bf{k}}\text{'},\,{\bf{p}}\text{'})$$

It is important to note, that the gauge transformation Eq. (18) ensures that the integrals of the kernels *P*_*νμ*_(**k**, **p**; **k′**, **p′**) over the first coordinates **k**, **p** are finite, which allows the interpretation of *P*_*νμ*_(**k**, **p**; **k′**, **p′**) as a transition rate from the state $|{\bf{k}}\text{'},\,{\bf{p}}\text{'},\,\mu \rangle$ to the state $|{\bf{k}},\,{\bf{p}},\,\nu \rangle$, and rewriting Eq. (19) in form of a master equation

20 $$\begin{array}{c}{g}_{\nu ,n+1}({\bf{k}},\,{\bf{p}})-{g}_{\nu ,n}({\bf{k}},\,{\bf{p}})=\sum _{\mu =\mathrm{0,2}}\,\int \,{d}^{2}{\bf{k}}\text{'}{d}^{2}{\bf{p}}\text{'}[{P}_{\nu \mu }({\bf{k}},\,{\bf{p}};\,{\bf{k}}\text{'},\,{\bf{p}}\text{'})\\ \,\,\,\,\,\,\,\,\,\,\,\,\,-\,{\delta }_{\nu \mu }\delta \,({\bf{k}}-{\bf{k}}\text{'})\delta \,({\bf{p}}-{\bf{p}}\text{'})]\,{g}_{\nu ,n}({\bf{k}}\text{'},\,{\bf{p}}\mathrm{\text{'}).}\end{array}$$

The kernels *P*_*νμ*_(**k**, **p**; **k**′ **p**′) are obtained straightforwardly from Eq. (14) and transformation Eq. (18). The bound four-atomic state is realized as a stationary solution of Eq. (20). To realize the numerical implementation of Eq. (19) as a stochastic Markovian evolution process, we need to interpret the transition rates in Eq. (20) as *probabilities* of a jump of a particle. To reach this, we divide RHS of Eq. (20) by a maximal value of the total escape rates

21 $${\Gamma }_{\mu }({\bf{k}}\text{'},\,{\bf{p}}\text{'})=\sum _{\nu =\mathrm{0,2}}\,\int \,{d}^{2}{\bf{k}}{d}^{2}{\bf{p}}{P}_{\nu \mu }({\bf{k}},\,{\bf{p}};\,{\bf{k}}\text{'},\,{\bf{p}}\text{'})$$

from the state $|{\bf{k}}\text{'},\,{\bf{p}}\text{'},\,\mu \rangle$. This operation is equivalent to the rescaling of the (discrete) time in Eq. (20), thus it does not change the stationary state we are interested in. The resulting equations read

22 $$\begin{array}{c}{g}_{\nu ,n+1}({\bf{k}},\,{\bf{p}})-{g}_{\nu ,n}({\bf{k}},\,{\bf{p}})=-{\gamma }_{\nu }({\bf{k}},\,{\bf{p}}){g}_{\nu ,n}({\bf{k}},\,{\bf{p}})\\ \,\,\,\,\,\,\,\,\,\,\,\,+\sum _{\nu ^{\prime} }\,\int \,{d}^{2}k\text{'}{d}^{2}p\text{'}\{{W}_{\nu \nu ^{\prime} }({\bf{k}},\,{\bf{p}};\,{\bf{k}}\text{'},\,{\bf{p}}\text{'}){g}_{\nu ^{\prime} ,n}({\bf{k}}\text{'},\,{\bf{p}}\text{'})\\ \,\,\,\,\,\,\,\,\,\,\,\,-{W}_{\nu ^{\prime} \nu }({\bf{k}}\text{'},\,{\bf{p}}\text{'};\,{\bf{k}},\,{\bf{p}}){g}_{\nu ,n}({\bf{k}},\,{\bf{p}})\},\end{array}$$

where

23 $${W}_{\nu \nu ^{\prime} }({\bf{k}},\,{\bf{p}};\,{\bf{k}}\text{'},\,{\bf{p}}\text{'})={P}_{\nu \nu ^{\prime} }({\bf{k}},\,{\bf{p}};\,{\bf{k}}\text{'},\,{\bf{p}}\text{'})/C,$$

24 $${\gamma }_{\nu }({\bf{k}},\,{\bf{p}})=\frac{1}{C}(1-{{\rm{\Gamma }}}_{\nu }({\bf{k}},\,{\bf{p}})-\frac{\lambda }{\mathrm{ln}\,\mathrm{[(2}-\varepsilon \mathrm{)(1}+{k}^{2}+{p}^{2})]}{\delta }_{\nu 2}),$$

and

25 $$C > \mathop{max}\limits_{\{{\bf{k}},{\bf{p}}\}}\{{{\rm{\Gamma }}}_{2}({\bf{k}},\,{\bf{p}})+\frac{\lambda }{\mathrm{ln}\,\mathrm{[(2}-\varepsilon \mathrm{)(1}+{k}^{2}+{p}^{2})]},\,{{\rm{\Gamma }}}_{0}({\bf{k}},\,{\bf{p}})\}.$$

For practical calculations in the region −1 < *ε* ≤ 0, 0 < *λ* < 2, *C* = 20 is the optimal choice. The choice of the factor *C* garanties

26 $$\sum _{\nu =\mathrm{0,2}}\,\int \,{d}^{2}{\bf{k}}{d}^{2}{\bf{p}}{W}_{\nu \nu ^{\prime} }({\bf{k}},\,{\bf{p}};\,{\bf{k}}\text{'},\,{\bf{p}}\text{'}) < \mathrm{1,}$$

which allows the interpretation of *W*_*νν*′_(**k**, **p**; **k**′, **p**′) as a probability density for the jump of the particle out of the state $|{\bf{k}}\text{'},\,{\bf{p}}\text{'},\,\nu ^{\prime} \rangle$ into the state $|{\bf{k}},\,{\bf{p}},\,\nu \rangle$. Now we can formulate Markovian stochastic process, which is described by the master equation Eq. (22) as follows: Consider a ensemble of walkers that evolve in the 4-dimensional space *k* = (**k**, **p**) and have an intrinsic flavor *ν* = 0, 2. At each discrete time step a walker in the state $|{\bf{k}},\,{\bf{p}},\,\nu \rangle$ is subject to the following elementary process: (i) jump to the state $|{\bf{k}}\text{'},\,{\bf{p}}\text{'},\,\nu ^{\prime} \rangle$ with the probability *W*_*ν*′*ν*_(**k**′, **p**'; **k**, **p**) by changing the flavor to *ν*′ (the same flavor is kept if *ν*′ = *ν*); (ii) the walker is destroyed with the probability *γ*_*ν*_(**k**, **p**). If *γ*_*ν*_(**k**, **p**) < 0, another walker is created in the state $|{\bf{k}},\,{\bf{p}},\,\nu \rangle$ with the probability |*γ*_*ν*_(**k**, **p**)|.

Implementing that algorithm numerically, the stationary solution is distinguished by a total number of walkers fluctuating around a stable mean value. Generically, due to finite probabilities *γ*_*ν*_(**k**) either all initially created walkers die out, or their number grows unbounded. The final outcome of the evolution is crucially affected by the term *λ*/ln(2 − *ε* + *k*^2^ + *p*^2^) that governs creation or annihilation of walkers in the *ν* = 2 channel. For a generic value of *λ*, the total number of walkers grows without bounds for small |*ε*|, and decays to zero after |*ε*| exceeds some critical value, that corresponds to the energy of the bound 4-atomic state. In the numerical procedure, the value of *ε* is adjusted to reach the situation with stationary average number of walkers. Details of numerical implementation and the link to the Mathematica notebook file are given in the Supplementary Information.

Numerical procedure was performed for 20 independent runs with *λ* varying from 0.8 to 2.0. The values of *ε* < 0 that stabilize the total number of walkers could be detected for *λ* > 1.4. For each *λ*, the average value of *ε* over the 20 runs was taken for the plot in Fig. 1, the standard deviation of the average determined the error bar.

## Electronic supplementary material

Supplementary Information

## Data Availability

The Mathematica notebook file generating all data analyzed during this study is available under the link https://drive.google.com/open?id=11wdTyWEzRgTX8B2DEqJe9jURr7z9o0JV.
