# Supplementary material for: Symmetry-restoring quantum phase transition in a two-dimensional spinor condensate
Source: Sci Rep. 2018 Aug 20;8:12468. doi: 10.1038/s41598-018-30876-x (PMC6102292; doi:10.1038/s41598-018-30876-x)
Supplement: Supplementary file 1 — Supplementary Information [file 41598_2018_30876_MOESM1_ESM.pdf]

Symmetry-restoring quantum phase transition in a two-dimensional spinor condensate. Supplementary material.

A. L. Chudnovskiy and V. Cheianov

June 18, 2018

In this supplementary material we provide details of the derivation of the main formulas in the text of the paper.

1 Two particle scattering amplitude from the Bethe-Peierls boundary conditions in two dimensions

Consider a collision of two particles with mass 1 in 2D. We are interested in s-wave scattering. The wave function as a function of the the relative coordinate satisfies the Schrödinger equation

$$\left\{ \left(\frac{d^2}{dr^2} + \frac{1}{r}\frac{d}{dr} \right) + 2\mu(E - V(r)) \right\} \psi(r) = 0. \tag{1}$$

Here μ is the reduced mass, $\mu = 1/2$. If $V(r)$ is a deep finite range potential, it can be emulated by the Bethe-Peierls boundary condition, which in 2D is formulated on a circle of small compared to the de Broglie wave length radius R_0. The Bethe-Peierls boundary condition reads

$$\left. \frac{d\psi/dr}{\psi} \right|_{r=R_0} = -\frac{1}{a}. \tag{2}$$

The solution of the scattering problem can be written in terms of the Green function describing a free motion of particles in 2D space as follows

$$\psi(\mathbf{r}, t) = \psi_0(\mathbf{r}, t) + \int d^2\mathbf{r}' G(\mathbf{r} - \mathbf{r}')\delta(|\mathbf{r} - \mathbf{r}'| - R_0) f(\mathbf{r}'). \tag{3}$$

The function ψ_0 describes the incoming plane wave. The Green function describes the scattered wave, and the integration goes over the region, where the scattering takes place. The Green function satisfies the equation

$$\left(-\frac{1}{2\mu}\nabla^2 - E \right) G(\mathbf{r}, \mathbf{r}') = \delta(\mathbf{r} - \mathbf{r}'). \tag{4}$$

Introduce the parameter $k = \sqrt{2\mu|E|}$, and the rescaled coordinate $\mathbf{x} = k\mathbf{r}$. Then for $E > 0$ the scattered wave is described by the function $G(|\mathbf{r} - \mathbf{r}'|) = CH_0^{(1)}(|\mathbf{r} - \mathbf{r}'|)$, where $H_0^{(1)}(x)$ denotes the Hankel function. The constant C is determined by substitution in Eq. (4) and integrating over the circle $|\mathbf{r} - \mathbf{r}'| = R_0$. Using the short range approximation $H_0^{(1)}(x) \approx \frac{2i}{\pi} \ln x + 1$, we get $C = \frac{i\mu}{2}$ and hence

$$G(x) = \frac{i\mu}{2} H_0^{(1)}(x). \tag{5}$$

For $E < 0$ the scattered wave is described by $G(x) = CK_0(x)$, where $K_0(x)$ is the modified Bessel function of the second kind. Using the short-range asymptote $K_0(x) \approx -\ln x$, we fix the constant $C = \mu/\pi$ and obtain ($x = \sqrt{2\mu|E|}r$), $E < 0$

$$G_E(x) = \frac{\mu}{\pi} K_0(x). \tag{6}$$

Now we fix the function $f(\mathbf{r})$ by substituting the formal solution Eq. (3) in the boundary condition Eq. (2). Thereby the action of the scattering potential is replaced by a function $f(\mathbf{r}')$ on the circle of a small radius R_0. We obtain two equations, the one for the wave function and the one for its derivative

$$\psi(\mathbf{r}, t) = \psi_0(\mathbf{r}, t) + \oint_{|r'|=R_0} d\mathbf{l}' G(\mathbf{r} - R_0) f(R_0), \tag{7}$$

$$\partial_r \psi(\mathbf{r}, t) = \partial_r \psi_0(\mathbf{r}, t) + \oint_{|r'|=R_0} d\mathbf{l}' \partial_r G(\mathbf{r} - R_0) f(R_0). \tag{8}$$

s-wave scattering implies the angular independence of $f(R_0)$, which allows to put it out of the integration. Furthermore, at small distances (correspondingly large wave vectors), one can neglect the energy in Eq. (4) for the Green function. In that way the equation for the Green function acquires the form of the equation for the Coulomb potential in 2D. Putting $\mu = 1/2$, we get

$$\nabla^2 G(\mathbf{r}, \mathbf{r}') = -\delta(\mathbf{r} - \mathbf{r}'). \tag{9}$$

The condition for that approximation reads

$$\sqrt{2\mu|E|} R_0 \ll 1, \tag{10}$$

which determines the small parameter in the following derivations. Furthermore, using the Gauss theorem, we can understand the integral over the circle as a potential created by the homogeneous charge distribution on the circle, which in turn is equal to the potential of the total charge places in the center of the circle. It follows that the result of the integration does not change if we replace the argument R_0 by zero in the Green function. Applying this line of arguments we obtain

$$\oint_{|r'|=R_0} G_E(\mathbf{r} - \mathbf{r}') f(R_0) d\mathbf{l} = f(R_0) \oint_{|r'|=R_0} G_E(\mathbf{r} - \mathbf{R_0}) d\mathbf{l} = 2\pi R_0 f(R_0) G_E(r). \tag{11}$$

Now we can evaluate integrals in in Eqs. (7), (8) using Eq. (11). Furthermore, since the derivative of the incoming wave $\partial_r \psi_0$ is a smooth function at small r whereas the Green function develops a singularity, one can neglect the term $\partial_r \psi_0$ in Eq. (8). Then the boundary condition assumes the form

$$\frac{\partial_r \psi}{\psi}|_{r=R_0} = \frac{2\pi R_0 f(R_0) \partial_r G_E(R_0)}{\psi_0(R_0) + 2\pi R_0 f(R_0) G_E(R_0)} = -\frac{1}{a}. \tag{12}$$

Using the asymptote of Green function at small r, and replacing $\psi_0(R_0) \approx 1$, we solve Eq. (12) with respect to $f(R_0)$. The solution the form

$$f(R_0) = \frac{1}{2\mu R_0 \left[\ln\left(kR_0 e^{a/R_0}\right) - i\pi/2\right]}, \text{ for } E > 0, \tag{13}$$

$$f(R_0) = \frac{1}{2\mu R_0 \ln\left(kR_0 e^{a/R_0}\right)}, \text{ for } E < 0. \tag{14}$$

Furthermore, in the case $E > 0$ we can relate $f(R_0)$ to the scattering amplitude. Substituting $f(R_0)$ in the general solution Eq. (3), we obtain for $E > 0$

$$\psi(\mathbf{r}, t) = \psi_0(\mathbf{r}, t) + i\pi\mu R_0 f(R_0) H_0^{(1)}(kr). \tag{15}$$

Using the large distance asymptote

$$H_0^{(1)}(x) = \sqrt{\frac{2}{\pi x}} e^{ix} e^{-i\pi/4} \tag{16}$$

we write down Eq. (15) in the form

$$\psi(\mathbf{r},t) = \psi_0(\mathbf{r},t) + e^{i\frac{\pi}{4}} \sqrt{\frac{2\pi}{k}} \mu R_0 f(R_0) \frac{e^{ikr}}{\sqrt{r}}, \tag{17}$$

from which we identify the scattering amplitude as

$$A = \sqrt{\frac{2\pi}{k}} \mu R_0 f(R_0) = \frac{\sqrt{\pi}}{\sqrt{2k}\left[\ln\left[kR_0 e^{\frac{a}{R_0}}\right] - i\pi/2\right]}, \tag{18}$$

where we put $\mu = 1/2$. The continuation of the scattering amplitude to negative energies is obtained by using the expression (14) in Eq. (19), which results in

$$A = \frac{\sqrt{\pi}}{\sqrt{2k}\ln\left[kR_0 e^{\frac{a}{R_0}}\right]}, \tag{19}$$

For $a > 0$, the scattering amplitude has a pole as a function of the energy ($k = \sqrt{|E|}$) at

$$E = -|E| = -\frac{1}{R_0^2} e^{-2\frac{a}{R_0}}, \tag{20}$$

which corresponds to the formation of a bound molecular state. For $a < 0$, the scattering amplitude remains negative for all energies without showing any resonant structure.

To obtain the relation of the scattering parameter a with the s-wave scattering length in 3 dimensions, we compare Eq. (19) with the expressions for the scattering amplitudes in presence of confinement potential derived in Refs. [1, 2]. The comparison results in the following equation

$$\ln\left[kR_0 e^{\frac{a}{R_0}}\right] = -\sqrt{\frac{\pi}{2}} \frac{\ell_0}{a_{3D}} + \ln\left(k\ell_0 \sqrt{\frac{\pi}{2B}}\right). \tag{21}$$

Equating k-dependent and k-independent parts of Eq. (21), we obtain

$$\frac{a}{R_0} = -\sqrt{\frac{\pi}{2}} \frac{\ell_0}{a_{3D}}, \; R_0 = \ell_0 \sqrt{\frac{\pi}{2B}}, \tag{22}$$

which constitutes Eq. (3) of the main text of the paper.

2 Structure of the wave function for scattering of two singlet pairs

We consider scattering of two singlet bound states, which we also call molecules. The total spin of the four-particle state equals 0. We only consider the elastic scattering events, therefore the out-state still consists of the two singlet molecules. Guided by that reason, we introduce the basis $\mathcal{B} = \{\Phi_1, \Phi_2, \Phi_3\}$ in the spin-0 subspace of the spin Hilbert space of the four atoms as follows

$$\Phi_1 = |1,4\rangle_s \otimes |2,3\rangle_s, \; \Phi_2 = |2,4\rangle_s \otimes |1,3\rangle_s, \; \Phi_3 = |3,4\rangle_s \otimes |1,2\rangle_s. \tag{23}$$

Here $|i,j\rangle_s$ denotes the singlet state formed by the atoms (i,j), the index of the state Φ_i corresponds to the number of the atom that forms a singlet state with the atom 4. The general two-molecule wave function can now be written as

$$\Psi(\mathbf{r}_1,\mathbf{r}_2,\mathbf{r}_3,\mathbf{r}_4) = \chi_1(\mathbf{r}_1,\mathbf{r}_2,\mathbf{r}_3,\mathbf{r}_4)\Phi_1 + \chi_2(\mathbf{r}_1,\mathbf{r}_2,\mathbf{r}_3,\mathbf{r}_4)\Phi_2 + \chi_3(\mathbf{r}_1,\mathbf{r}_2,\mathbf{r}_3,\mathbf{r}_4)\Phi_3. \tag{24}$$

Here χ_i describes the spatial part of the wave function and Φ_i relates to the spin part, and $\mathbf{r}_j$, $j=1,2,3,4$ is the coordinate of the j's atom.

2.1 Representation of permutation operators in the basis $\mathcal{B}$

Direct calculation shows that in the basis (Φ_1,Φ_2,Φ_3) the permutation operators are represented by

$$\Pi_{12} = \Pi_{34} = \begin{pmatrix} 0 & 1 & 0 \\ 1 & 0 & 0 \\ 0 & 0 & 1 \end{pmatrix}, \tag{25}$$

$$\Pi_{13} = \Pi_{24} = \begin{pmatrix} 0 & 0 & 1 \\ 0 & 1 & 0 \\ 1 & 0 & 0 \end{pmatrix}, \tag{26}$$

$$\Pi_{14} = \Pi_{23} = \begin{pmatrix} 1 & 0 & 0 \\ 0 & 0 & 1 \\ 0 & 1 & 0 \end{pmatrix}. \tag{27}$$

Representations of other permutation operators are obtained according to the obvious relation $\Pi_{ij} = \Pi_{ji}$.

2.2 Projectors on the $F=0$ and $F=2$ scattering channels in the basis $\mathcal{B}$

We denote $\hat{P}_{ij}^{(\nu)}$ the projection operator on the subspace, in which the atoms i,j have the total spin ν, $(\nu=0,2)$. Explicit form of the projector onto $F=0$ state in terms of spin operators can be written as

$$\hat{P}_{ij}^{(0)} = \frac{1}{12}[(\hat{\mathbf{S}}_i+\hat{\mathbf{S}}_j)^2-6][(\hat{\mathbf{S}}_i+\hat{\mathbf{S}}_j)^2-2]. \tag{28}$$

Using Eq. (28), and definition Eq. (23), we obtain the following matrix presentation of the projectors in the basis $\mathcal{B}$

$$\hat{P}_{12}^{(0)} = \begin{pmatrix} 0 & 0 & 0 \\ 0 & 0 & 0 \\ \frac{1}{3} & \frac{1}{3} & 1 \end{pmatrix}, \ \hat{P}_{23}^{(0)} = \begin{pmatrix} 1 & \frac{1}{3} & \frac{1}{3} \\ 0 & 0 & 0 \\ 0 & 0 & 0 \end{pmatrix}, \ \hat{P}_{31}^{(0)} = \begin{pmatrix} 0 & 0 & 0 \\ \frac{1}{3} & 1 & \frac{1}{3} \\ 0 & 0 & 0 \end{pmatrix}. \tag{29}$$

According to the construction of the basis states, the singlet state of the atom 4 and the atom i means also the singlet of the two complementary atoms, j and k, where $j,k \neq i,4$. Therefore, for the projectors involving the atom 4, we have

$$\hat{P}_{14}^{(0)} = \hat{P}_{23}^{(0)}, \ \hat{P}_{24}^{(0)} = \hat{P}_{13}^{(0)}, \ \hat{P}_{34}^{(0)} = \hat{P}_{12}^{(0)}. \tag{30}$$

Explicit form of the projector onto $F=2$ state in terms of spin operators can be written as

$$\hat{P}_{ij}^{(2)} = \frac{1}{24}(\hat{\mathbf{S}}_i+\hat{\mathbf{S}}_j)^2[(\hat{\mathbf{S}}_i+\hat{\mathbf{S}}_j)^2-2]. \tag{31}$$

The matrix representation of the projection operator $\hat{P}_{12}^{(2)}$ is given by

$$\hat{P}_{12}^{(2)} = \frac{1}{6}\begin{pmatrix} 3 & 3 & 0 \\ 3 & 3 & 0 \\ -2 & -2 & 0 \end{pmatrix}. \tag{32}$$

Other projection operators $P_{ij}^{(2)}$ are obtained by action of the permutation operator on $\hat{P}_{12}^{(2)}$ according to the rule

$$P_{ij}^{(2)} = \Pi_{1i}\Pi_{2j}P_{12}^{(2)}\Pi_{1i}\Pi_{2j} \tag{33}$$

2.3 Derivation of STM equations

Now the Bethe-Peierls boundary conditions can be formulated for each pair of particles i, j as

$$\left(\partial_{r_{ij}}\hat{P}_{ij}^{(\nu)}\boldsymbol{\chi} + \frac{1}{a_\nu}\hat{P}_{ij}^{(\nu)}\boldsymbol{\chi}\right)\Bigg|_{|\mathbf{r}_{ij}|=R_0} = 0, \tag{34}$$

where $\boldsymbol{\chi} = (\chi_1, \chi_2, \chi_3)^T$. The boundary conditions Eq. (34) are formulated on the circle $r_{ij} = R_0$. The general solution in terms of Green's function can be written as (cf. Eq. (3))

$$\boldsymbol{\chi} = \boldsymbol{\chi}_0 + \sum_{\langle i,j\rangle}\int_{|\mathbf{r}'_i-\mathbf{r}'_j|=R_0} G_E(\mathbf{X}-\mathbf{X}')\boldsymbol{f}^{ij}(\mathbf{X}')d\mathbf{X}', \tag{35}$$

where

$$\left(\frac{1}{2}\nabla^2_{\mathbf{X}} + E\right)G_E(\mathbf{X}-\mathbf{X}') = -\delta(\mathbf{X}-\mathbf{X}'), \tag{36}$$

and we introduced 8-dimensional coordinate vectors

$$\mathbf{X} = (\mathbf{r}_1, \mathbf{r}_2, \mathbf{r}_3, \mathbf{r}_4), \;\; \mathbf{X}' = (\mathbf{r}'_1, \mathbf{r}'_2, \mathbf{r}'_3, \mathbf{r}'_4). \tag{37}$$

In Eq. (35) we introduced a vector-valued function $\boldsymbol{f}^{ij} = (f_1^{ij}, f_2^{ij}, f_3^{ij})^T$ for each pair of points (ij) with its domain given by the cylinder $r'_{ij} = R_0$.

The symmetry of the wave function under permutations of atoms induces linear relationships between the functions $\boldsymbol{f}^{ij}$.

$$\hat{\Pi}_{ij}\boldsymbol{f}^{ij} = \boldsymbol{f}^{ij}, \tag{38}$$

$$\hat{\Pi}_{ij}\boldsymbol{f}^{jk} = \boldsymbol{f}^{ik}, \tag{39}$$

where we suppressed the spatial arguments. Note, that no summation over repeating indexes is implied in Eqs. (38), (39). For $\boldsymbol{f}^{12}$ Eq. (38) implies

$$f_1^{12} = f_2^{12}, \;\; f_2^{12} = f_1^{12}. \tag{40}$$

It follows that the function $\boldsymbol{f}^{12}(\mathbf{X})$ can be parametrized by two independent functions $\alpha(\mathbf{X})$ and $\beta(\mathbf{X})$

$$\boldsymbol{f}^{12} = \alpha\begin{pmatrix}1\\1\\0\end{pmatrix} + \beta\begin{pmatrix}0\\0\\1\end{pmatrix}. \tag{41}$$

Applying the relations Eqs. (39) to Eq. (41), we obtain $\boldsymbol{f}^{34} = \boldsymbol{f}^{12}$, and

$$\boldsymbol{f}^{23} = \boldsymbol{f}^{14} = \alpha\begin{pmatrix}0\\1\\1\end{pmatrix} + \beta\begin{pmatrix}1\\0\\0\end{pmatrix}, \tag{42}$$

$$\boldsymbol{f}^{13} = \boldsymbol{f}^{24} = \alpha\begin{pmatrix}1\\0\\1\end{pmatrix} + \beta\begin{pmatrix}0\\1\\0\end{pmatrix}. \tag{43}$$

In terms of the functions $\alpha(\mathbf{X})$ and $\beta(\mathbf{X})$, the general solution for the wave function acquires the form

$$\begin{pmatrix} \chi_1(\mathbf{X}) \\ \chi_2(\mathbf{X}) \\ \chi_3(\mathbf{X}) \end{pmatrix} = \begin{pmatrix} \chi_1^0(\mathbf{X}) \\ \chi_2^0(\mathbf{X}) \\ \chi_3^0(\mathbf{X}) \end{pmatrix} + \left\{ \left(\int_{|\mathbf{r}'_1-\mathbf{r}'_2|=R_0} + \int_{|\mathbf{r}'_3-\mathbf{r}'_4|=R_0} \right) \begin{pmatrix} \alpha(\mathbf{X}') \\ \alpha(\mathbf{X}') \\ \beta(\mathbf{X}') \end{pmatrix} \right.$$
$$\left. + \left(\int_{|\mathbf{r}'_1-\mathbf{r}'_3|=R_0} + \int_{|\mathbf{r}'_2-\mathbf{r}'_4|=R_0} \right) \begin{pmatrix} \alpha(\mathbf{X}') \\ \beta(\mathbf{X}') \\ \alpha(\mathbf{X}') \end{pmatrix} + \left(\int_{|\mathbf{r}'_1-\mathbf{r}'_4|=R_0} + \int_{|\mathbf{r}'_2-\mathbf{r}'_3|=R_0} \right) \begin{pmatrix} \beta(\mathbf{X}') \\ \alpha(\mathbf{X}') \\ \alpha(\mathbf{X}') \end{pmatrix} \right\} G_E(\mathbf{X}-\mathbf{X}')d\mathbf{X}', \tag{44}$$

Now we apply the boundary condition Eq. (34) to the general form Eq. (44), and derive equations for the functions α and β. For instance, the application of the boundary condition Eq. (34) at $|\mathbf{r}_1 - \mathbf{r}_2| = R_0$ for $\nu = 2$ channel leads to equation

$$\partial_{r_{12}}(\chi_1^0 + \chi_2^0)\Big|_{|\mathbf{r}_{12}|=R_0} + 2\partial_{r_{12}} \left\{ \left[\int_{|\mathbf{r}'_{12}|=R_0} + \int_{|\mathbf{r}'_{34}|=R_0} \right] G_E(\mathbf{X}-\mathbf{X}')\alpha(\mathbf{X}')d\mathbf{X}' + \right.$$
$$\left. \left[\int_{|\mathbf{r}'_{13}|=R_0} + \int_{|\mathbf{r}'_{14}|=R_0} + \int_{|\mathbf{r}'_{23}|=R_0} + \int_{|\mathbf{r}'_{24}|=R_0} \right] G_E(\mathbf{X}_i - \mathbf{X}'_i)\left(\alpha(\mathbf{X}') + \beta(\mathbf{X}')\right) d\mathbf{X}' \right\} \Big|_{|\mathbf{r}_{12}|=R_0} =$$
$$-\frac{1}{a_2} \left\{ (\chi_1^0 + \chi_2^0)\Big|_{|r_{12}|=R_0} + 2 \left[\int_{|\mathbf{r}'_{12}|=R_0} + \int_{|\mathbf{r}'_{34}|=R_0} \right] G_E(\mathbf{X}-\mathbf{X}')\alpha(\mathbf{X}')d\mathbf{X}' + \right.$$
$$\left. \left[\int_{|\mathbf{r}'_{13}|=R_0} + \int_{|\mathbf{r}'_{14}|=R_0} + \int_{|\mathbf{r}'_{23}|=R_0} + \int_{|\mathbf{r}'_{24}|=R_0} \right] G_E(\mathbf{X}-\mathbf{X}')\left(\alpha(\mathbf{X}') + \beta(\mathbf{X}')\right) d\mathbf{X}' \right\} \Big|_{|\mathbf{r}_{12}|=R_0}.$$

On the left hand side one can leave only the most singular term for $r_{12} \to R_0$, in which the the derivative of the Green function is taken by the variable r_{12}, normal to the scattering surface at which the boundary condition is imposed. We obtain

$$\int_{|\mathbf{r}'_{12}|=R_0} \partial_{r_{12}} G_E(\mathbf{X}_i - \mathbf{X}'_i)\left(2\alpha(\mathbf{X}')\right) d\mathbf{X}' \Big|_{|\mathbf{r}_{12}|=R_0} = \mathcal{I}_0^{(2)} - \frac{1}{a_2} \left\{ \left[\int_{|\mathbf{r}'_{12}|=R_0} + \int_{|\mathbf{r}'_{34}|=R_0} \right] G_E(\mathbf{r}_i - \mathbf{r}'_i)(2\alpha(\mathbf{X}'))d\mathbf{X}' + \right.$$
$$\left. \left[\int_{|\mathbf{r}'_{13}|=R_0} + \int_{|\mathbf{r}'_{14}|=R_0} + \int_{|\mathbf{r}'_{23}|=R_0} + \int_{|\mathbf{r}'_{24}|=R_0} \right] G_E(\mathbf{X}-\mathbf{X}')\left(\alpha(\mathbf{X}') + \beta(\mathbf{X}')\right) d\mathbf{X}' \right\} \Big|_{|\mathbf{r}_{12}|=R_0}. \tag{45}$$

Here

$$\mathcal{I}_0^{(2)} = -\left(\frac{1}{a_2} + \partial_{r_{12}} \right) (\chi_1^0 + \chi_2^0)\Big|_{|\mathbf{r}_{12}|=R_0} \tag{46}$$

denotes the source field, describing the incoming wave in the $F = 2$ channel. Analogously, in the $F = 0$ channel, we obtain

$$\int_{|\mathbf{r}'_{12}|=R_0} \partial_{r_{12}} G_E(\mathbf{X}-\mathbf{X}') \left(\frac{2}{3}\alpha(\mathbf{X}') + \beta(\mathbf{X}') \right) d\mathbf{X}' \Big|_{|\mathbf{r}_{12}|=R_0} =$$
$$\mathcal{I}_0^{(0)} - \frac{1}{a_0} \left\{ \left[\int_{|\mathbf{X}'_{12}|=R_0} + \int_{|\mathbf{X}'_{34}|=R_0} \right] G_E(\mathbf{X}_i - \mathbf{X}'_i) \left(\frac{2}{3}\alpha(\mathbf{X}') + \beta(\mathbf{X}') \right) d\mathbf{X}' + \right.$$
$$\left. \left[\int_{|\mathbf{r}'_{13}|=R_0} + \int_{|\mathbf{r}'_{14}|=R_0} + \int_{|\mathbf{r}'_{23}|=R_0} + \int_{|\mathbf{r}'_{24}|=R_0} \right] G_E(\mathbf{X}-\mathbf{X}') \left(\frac{4}{3}\alpha(\mathbf{X}') + \frac{1}{3}\beta(\mathbf{X}') \right) d\mathbf{X}' \right\} \Big|_{|\mathbf{r}_{12}|=R_0}, \tag{47}$$

where

$$\mathcal{I}_0^{(0)} = -\left(\frac{1}{a_2} + \partial_{r_{12}}\right)\left[\frac{1}{3}(\chi_1^0 + \chi_2^0) + \chi_3^0\right]\Bigg|_{|\mathbf{r}_{12}|=R_0} \tag{48}$$

denotes the source field, describing the incoming wave in the $F = 0$ channel. Due to the permutation symmetry, the boundary conditions at other pairs of points $|\mathbf{r}_{ij}| = R_0$ do not lead to new independent equations.

2.3.1 Transition to relative coordinates and separation of singularity

The center of mass coordinate of four atoms is given by $\mathbf{R} = \frac{1}{4}(\mathbf{r}_1 + \mathbf{r}_2 + \mathbf{r}_3 + \mathbf{r}_4)$. For the following calculations we introduce the set of relative (Jacobi) coordinates:

$$\mathbf{z} = (\mathbf{r}_3 - \mathbf{r}_4), \quad \mathbf{y} = (\mathbf{r}_1 - \mathbf{r}_2), \quad \mathbf{x} = \frac{1}{\sqrt{2}}[(\mathbf{r}_3 + \mathbf{r}_4) - (\mathbf{r}_1 + \mathbf{r}_2)]. \tag{49}$$

The boundary condition at $|\mathbf{r}_{12}| = R_0$ now acquires the form $|\mathbf{y}| = R_0$. We go to the center of mass system by integrating Eqs. (45), (47) over the center of mass coordinate $\mathbf{R}$. The resulting equations depend only on the relative coordinates given by Eq. (49). The free four-particle Green function in relative coordinates satisfies the equation

$$(\nabla_{\mathbf{x}}^2 + \nabla_{\mathbf{y}}^2 + \nabla_{\mathbf{z}}^2 + E)G_E(\mathbf{x} - \mathbf{x}', \mathbf{y} - \mathbf{y}', \mathbf{z} - \mathbf{z}') = -2\delta(\mathbf{x} - \mathbf{x}')\delta(\mathbf{y} - \mathbf{y}')\delta(\mathbf{z} - \mathbf{z}'), \tag{50}$$

which is an equation for a Green function of a free particle in 6 dimensions. In the case of negative energy $E = -|E| < 0$ the solution of Eq. (50) can be written in the form

$$G_E(\mathbf{Z}) = |E|^2 G_0(\sqrt{|E|}|\mathbf{Z}|), \tag{51}$$

where

$$G_0(\xi) = \frac{K_2(\xi)}{4\pi^3\xi^2}, \tag{52}$$

and $\mathbf{Z} = (\mathbf{x}, \mathbf{y}, \mathbf{z})$ is the 6-dimensional vector of relative coordinates. $K_2(\xi)$ denotes the modified Bessel function. The Fourier transformed Green function is given by

$$G_0(\mathbf{K}) = \frac{2}{|\mathbf{K}|^2 + 1}, \tag{53}$$

where $\mathbf{K} = (\mathbf{k_x}, \mathbf{k_y}, \mathbf{k_z})$ is the 6-dimensional wave vector.

To simplify the form of STM equations further, we introduce the source fields corresponding specifically to $S = 0$ and $S = 2$ channels as follows

$$f_0(\mathbf{Z}) = 2\pi R_0\left(\frac{2}{3}\alpha(\mathbf{Z}) + \beta(\mathbf{Z})\right), \; f_2(\mathbf{Z}) = 4\pi R_0\alpha(\mathbf{Z}). \tag{54}$$

The boundary $|\mathbf{r}_{12}| = R_0$ transforms in the relative coordinates to $|\mathbf{y}| = R_0$. Eqs. (45), (47) develop singularities at the surfaces $|\mathbf{r}'_{12}| = |\mathbf{r}_{12}| = R_0$, which in relative coordinates transforms to $|\mathbf{y}| = |\mathbf{y}'| = R_0$, when the collision surface coincides with the surface at which the boundary condition is imposed. The singularities are dealt with by subtraction and addition of $f_2(\mathbf{x}, R_0, \mathbf{z})$ or $f_0(\mathbf{x}, R_0, \mathbf{z})$ for Eqs. (45), (47) respectively, similarly to the procedure described in Ref. [3]. Finally, we introduce dimensionless coordinates by re-scaling $\mathbf{x} \to \mathbf{x}/\sqrt{|E|}$.

Then the STM equations acquire the following explicit form

$$\begin{aligned}
&\frac{f_0(\mathbf{x},\mathbf{z})}{\gamma_0} = \frac{1}{3}(\chi_1^0(\mathbf{x},0,\mathbf{z}) + \chi_2^0(\mathbf{x},0,\mathbf{z})) + \chi_3^0(\mathbf{x},0,\mathbf{z}) + \\
&\int [f_0(\mathbf{x}',\mathbf{z}')) - f_0(\mathbf{x},\mathbf{z}))] G_0(\mathbf{x}-\mathbf{x}',0,\mathbf{z}-\mathbf{z}') d^2\mathbf{x}' d^2\mathbf{z}' + \\
&\int G_0\left(\sqrt{(\mathbf{x}+\mathbf{x}')^2 + \mathbf{y}'^2 + \mathbf{z}^2}\right) f_0(\mathbf{x}',\mathbf{y}') d\mathbf{x}' d\mathbf{y}' + \\
&2\int \left[G_0\left(\sqrt{\mathbf{z}^2 + \mathbf{x}^2 + \sqrt{2}\mathbf{z}\cdot\mathbf{x}' + (\sqrt{2}\mathbf{x}-\mathbf{z})\cdot\mathbf{z}' + \mathbf{x}'^2 + \mathbf{z}'^2}\right) + \right. \\
&\left. G_0\left(\sqrt{\mathbf{z}^2 + \mathbf{x}^2 - \sqrt{2}\mathbf{z}\cdot\mathbf{x}' - (\sqrt{2}\mathbf{x}+\mathbf{z})\cdot\mathbf{z}' + \mathbf{x}'^2 + \mathbf{z}'^2}\right)\right] \left(\frac{1}{3} f_0(\mathbf{x}',\mathbf{z}') + \frac{5}{9} f_2(\mathbf{x}',\mathbf{z}')\right) d\mathbf{x}' d\mathbf{z}',
\end{aligned} \tag{55}$$

$$\begin{aligned}
&\frac{f_2(\mathbf{x},\mathbf{z})}{\gamma_2} = (\chi_1^0(\mathbf{x},0,\mathbf{z}) + \chi_2^0(\mathbf{x},0,\mathbf{z})) + \\
&\int [f_2(\mathbf{x}',\mathbf{z}') - f_2(\mathbf{x},\mathbf{z})] G_0(\mathbf{x}-\mathbf{x}',0,\mathbf{z}-\mathbf{z}') d^2\mathbf{x}' d^2\mathbf{z}' + \\
&\int G_0\left(\sqrt{(\mathbf{x}+\mathbf{x}')^2 + \mathbf{y}'^2 + \mathbf{z}^2}\right) f_2(\mathbf{x}',\mathbf{y}') d\mathbf{x}' d\mathbf{y}' + \\
&2\int G_0\left(\sqrt{\mathbf{z}^2 + \mathbf{x}^2 + \sqrt{2}\mathbf{z}\cdot\mathbf{x}' + (\sqrt{2}\mathbf{x}-\mathbf{z})\cdot\mathbf{z}' + \mathbf{x}'^2 + \mathbf{z}'^2}\right) \left(f_0(\mathbf{x}',\mathbf{z}') + \frac{1}{6} f_2(\mathbf{x}',\mathbf{z}')\right) d\mathbf{x}' d\mathbf{z}' + \\
&2\int G_0\left(\sqrt{\mathbf{z}^2 + \mathbf{x}^2 - \sqrt{2}\mathbf{z}\cdot\mathbf{x}' - (\sqrt{2}\mathbf{x}+\mathbf{z})\cdot\mathbf{z}' + \mathbf{x}'^2 + \mathbf{z}'^2}\right) \left(f_0(\mathbf{x}',\mathbf{z}') + \frac{1}{6} f_2(\mathbf{x}',\mathbf{z}')\right) d\mathbf{x}' d\mathbf{z}'.
\end{aligned} \tag{56}$$

where all distances are measured in units of $1/\sqrt{|E|}$. Here, all microscopic scattering parameters enter just in the form of two constants, γ_0 and γ_2, which are defined as follows

$$\gamma_0 = \frac{\pi}{\ln\left(R_0\sqrt{|E|}e^{a_0/R_0}\right)},\ \gamma_2 = \frac{\pi}{\ln\left(R_0\sqrt{|E|}e^{a_2/R_0}\right)}. \tag{57}$$

Fourier transform of Eqs. (55), (56) is performed according to the following Fourier representations of the Green functions

$$\begin{aligned}
&G_0\left(\sqrt{(\mathbf{x}+\mathbf{x}')^2 + \mathbf{y}'^2 + \mathbf{z}^2}\right) = \\
&\int \frac{d^2\mathbf{k}}{(2\pi)^2}\frac{d^2\mathbf{p}}{(2\pi)^2}\frac{d^2\mathbf{q}}{(2\pi)^2}\frac{2}{1+k^2+p^2+q^2} \exp\left[i\left\{\mathbf{k}\cdot\mathbf{z} + \mathbf{p}\cdot(\mathbf{x}+\mathbf{x}') - \mathbf{q}\cdot\mathbf{y}'\right\}\right],
\end{aligned} \tag{58}$$

and

$$\begin{aligned}
&G_0\left(\sqrt{\mathbf{z}^2 + \mathbf{x}^2 \pm \sqrt{2}\mathbf{z}\cdot\mathbf{x}' - (\mathbf{z}\mp\sqrt{2}\mathbf{x})\cdot\mathbf{z}' + \mathbf{x}'^2 + \mathbf{z}'^2}\right) = \\
&\int \frac{d^2\mathbf{k}}{(2\pi)^2}\frac{d^2\mathbf{p}}{(2\pi)^2}\frac{d^2\mathbf{q}}{(2\pi)^2}\frac{2}{1+k^2+p^2+q^2} \exp\left[i\left\{\mathbf{k}\cdot\frac{\mathbf{z}-\mathbf{z}'}{\sqrt{2}} + \mathbf{p}\cdot\left(\frac{\mathbf{z}}{\sqrt{2}} \pm \mathbf{x}'\right) + \mathbf{q}\cdot\left(\frac{\mathbf{z}'}{\sqrt{2}} \pm \mathbf{x}\right)\right\}\right].
\end{aligned} \tag{59}$$

Below we demonstrate the representation of two typical terms in Eqs. (55), (56) by Fourier components, from which the Fourier transform becomes obvious

$$\int G_0\left(\sqrt{(\mathbf{x}+\mathbf{x}')^2+\mathbf{y}'^2+\mathbf{z}^2}\right) f(\mathbf{x}',\mathbf{y}')d\mathbf{x}'d\mathbf{y}' =$$
$$\int \frac{d^2\mathbf{k}}{(2\pi)^2}\frac{d^2\mathbf{p}}{(2\pi)^2}\frac{d^2\mathbf{q}}{(2\pi)^2}\frac{2}{1+k^2+p^2+q^2}e^{i\mathbf{p}\cdot(\mathbf{x}+\mathbf{x}')}e^{-i\mathbf{q}\cdot\mathbf{y}'}e^{i\mathbf{k}\cdot\mathbf{z}}f(\mathbf{x}',\mathbf{y}') =$$
$$\int \frac{d^2\mathbf{k}}{(2\pi)^2}\frac{d^2\mathbf{p}}{(2\pi)^2}\frac{d^2\mathbf{q}}{(2\pi)^2}\frac{2}{1+k^2+p^2+q^2}f(-\mathbf{p},\mathbf{q})e^{i(\mathbf{p}\cdot\mathbf{x}+\mathbf{k}\cdot\mathbf{z})}. \tag{60}$$

This leads to the first term in the right hand side of Eq. (12) in the main text of the paper. Furthermore

$$\int G_0\left(\sqrt{\mathbf{z}^2+\mathbf{x}^2+\sqrt{2}\mathbf{z}\cdot\mathbf{x}'+(\sqrt{2}\mathbf{x}-\mathbf{z})\cdot\mathbf{z}'+\mathbf{x}'^2+\mathbf{z}'^2}\right) f(\mathbf{x}',\mathbf{z}') =$$
$$\int \frac{d^2\mathbf{k}}{(2\pi)^2}\frac{d^2\mathbf{p}}{(2\pi)^2}\frac{d^2\mathbf{q}}{(2\pi)^2}\frac{2}{1+k^2+p^2+q^2}\int\frac{d^2\mathbf{k}'}{(2\pi)^2}\frac{d^2\mathbf{q}'}{(2\pi)^2}\int d\mathbf{x}'d\mathbf{y}'f(\mathbf{k}',\mathbf{q}')e^{i\mathbf{x}'\cdot(\mathbf{k}'+\mathbf{p})}e^{i\mathbf{z}'\cdot\left(\mathbf{q}'+\frac{\mathbf{q}-\mathbf{k}}{\sqrt{2}}\right)}e^{i\mathbf{z}\cdot\left(\frac{\mathbf{k}+\mathbf{p}}{\sqrt{2}}\right)}e^{i\mathbf{x}\cdot\mathbf{q}} =$$
$$\int \frac{d^2\mathbf{k}}{(2\pi)^2}\frac{d^2\mathbf{p}}{(2\pi)^2}\frac{d^2\mathbf{q}}{(2\pi)^2}\frac{2}{1+k^2+p^2+q^2}f\left(-\mathbf{p},\frac{\mathbf{k}-\mathbf{q}}{\sqrt{2}}\right)e^{i\frac{\mathbf{k}+\mathbf{p}}{\sqrt{2}}\cdot\mathbf{z}}e^{i\mathbf{q}\cdot\mathbf{x}}. \tag{61}$$

Introducing new variables

$$\mathbf{P} = -\frac{\mathbf{q}+\mathbf{p}}{\sqrt{2}},\ \ \mathbf{Q} = \frac{\mathbf{q}-\mathbf{p}}{\sqrt{2}}, \tag{62}$$

we obtain

$$-\mathbf{p} = \frac{1}{\sqrt{2}}(\mathbf{P}+\mathbf{Q}),\ \ \frac{\mathbf{k}-\mathbf{q}}{\sqrt{2}} = \frac{1}{\sqrt{2}}(\mathbf{k}+\mathbf{P}-\mathbf{Q}), \tag{63}$$

which leads to the last term in the right hand side of Eq. (14) in the main text of the paper with $s=-1$.

3 STM-equations of the scattering of singlet molecule and an atom

STM-equations for the scattering of a singlet molecule and an atom are derived similarly to the case of two molecules. Their explicit form is given by

$$\left[\frac{1}{4\pi}\ln[(1-\epsilon)(1+k^2)]-\lambda\delta_{\nu 2}\right]f_\nu(\mathbf{k}) = \int\frac{d^2\mathbf{q}}{(2\pi)^2}\frac{1}{q^2+k^2+\mathbf{q}\cdot\mathbf{k}+\frac{3}{4}}\sum_{\mu=0,2}\mathcal{K}_{\nu\mu}f_\mu(\mathbf{q}). \tag{64}$$

Here the matrix $\mathcal{K}$ is given by

$$\mathcal{K} = \begin{pmatrix} 2/3 & 20/9 \\ 1 & 1/3 \end{pmatrix}. \tag{65}$$

The parameter ϵ denotes the deviation of the energy from the energy of the molecular bound state, and $\lambda = \frac{(a_0+|a_2|)}{\pi R_0}$, as is introduced in the main text of the paper.

We perform the gauge transformation, introducing the functions

$$g_\nu(\mathbf{k}) = \frac{\ln\left[(1-\varepsilon)(1+k^2)\right]}{1+k^2}f_\nu(\mathbf{k}),\ \ \nu=0,2. \tag{66}$$

Furthermore, assuming the functions $g_\nu(\mathbf{k})$ be independent of the direction of the vector $\mathbf{k}$ in view of the isotropy of the s-wave scattering, we integrate out the angular variable, thus arriving at one-dimensional integral equations for the functions $g_\nu(\xi)$, where $\xi = k^2$. The explicit form of those equations is given below

$$g_0(\xi) = \frac{2}{3}\frac{1}{1+\xi}\int_0^\infty d\eta\frac{g_0(\eta)+\frac{10}{3}g_2(\eta)}{\sqrt{(\xi+\eta+\frac{3}{4})^2-\xi\eta}}\frac{1+\eta}{\ln[(1-\epsilon)(1+\eta)]}, \tag{67}$$

$$g_2(\xi) = \frac{1}{1+\xi}\int_0^\infty d\eta \frac{g_0(\eta)+\frac{1}{3}g_2(\eta)}{\sqrt{(\xi+\eta+\frac{3}{4})^2-\xi\eta}}\frac{1+\eta}{\ln[(1-\epsilon)(1+\eta)]} - \frac{\lambda}{\ln[(1-\epsilon)(1+\xi)]}g_2(\xi). \tag{68}$$

In line of the procedure described in the main text of the paper, Eqs. (67), (68) were mapped on the stochastic Markovian evolution problem. Further numerical investigations revealed no three-particle bound states.

4 Details of numerical implementation of Markovian evolution

In this section we provide details of numerical implementation of the Markovian evolution process. Mathematica notebook script for numerical simulations is provided in Ref. [4].

Introducing the functions

$$g_\nu(\mathbf{k},\mathbf{p}) = f_\nu(\mathbf{k},\mathbf{p})\frac{\ln[(2-\epsilon)(1+k^2+p^2)]}{1+k^2+p^2}. \tag{69}$$

we rewrite Eq. (14) in the main text of the paper in a more explicit form

$$\begin{aligned}
g_0(\mathbf{k},\mathbf{p}) &= 4\pi\int\frac{d^2\mathbf{k}'}{(2\pi)^2}\frac{d^2\mathbf{p}'}{(2\pi)^2}\int\frac{d^2\mathbf{Q}}{(2\pi)^2}\frac{[1+(k')^2+(p')^2]}{\ln[(2+\epsilon)(1+(k')^2+(p')^2)]}\frac{1}{(1+k^2+p^2)(1+k^2+p^2+Q^2)}\times\\
&\{g_0(-\mathbf{k},\mathbf{Q})\delta(\mathbf{k}'+\mathbf{k})\,\delta(\mathbf{p}'-\mathbf{Q})+\\
&2\left[\frac{1}{3}g_0\left(\frac{\mathbf{p}+\mathbf{Q}}{\sqrt{2}},-\frac{\mathbf{k}+\mathbf{p}-\mathbf{Q}}{\sqrt{2}}\right)+\frac{5}{9}g_2\left(\frac{\mathbf{p}+\mathbf{Q}}{\sqrt{2}},-\frac{\mathbf{k}+\mathbf{p}-\mathbf{Q}}{\sqrt{2}}\right)\right]\delta\left(\frac{\mathbf{p}+\mathbf{Q}}{\sqrt{2}}-\mathbf{k}'\right)\delta\left(\frac{\mathbf{k}+\mathbf{p}-\mathbf{Q}}{\sqrt{2}}+\mathbf{p}'\right)\\
&+2\left[\frac{1}{3}g_0\left(\frac{\mathbf{p}-\mathbf{Q}}{\sqrt{2}},\frac{\mathbf{k}+\mathbf{p}+\mathbf{Q}}{\sqrt{2}}\right)+\frac{5}{9}g_2\left(\frac{\mathbf{p}-\mathbf{Q}}{\sqrt{2}},\frac{\mathbf{k}+\mathbf{p}+\mathbf{Q}}{\sqrt{2}}\right)\right]\delta\left(\frac{\mathbf{p}-\mathbf{Q}}{\sqrt{2}}-\mathbf{k}'\right)\delta\left(\frac{\mathbf{k}+\mathbf{p}+\mathbf{Q}}{\sqrt{2}}-\mathbf{p}'\right)\bigg\}.\\
g_2(\mathbf{k},\mathbf{p}) &= \frac{\lambda_2}{\ln[(2+\epsilon)(1+k^2+p^2)]}g_2(\mathbf{k},\mathbf{p})+\\
&4\pi\int\frac{d^2\mathbf{k}'}{(2\pi)^2}\frac{d^2\mathbf{p}'}{(2\pi)^2}\int\frac{d^2\mathbf{Q}}{(2\pi)^2}\frac{[1+(k')^2+(p')^2]}{\ln[(2+\epsilon)(1+(k')^2+(p')^2)]}\frac{1}{(2+\epsilon+k^2+p^2)(2+\epsilon+k^2+p^2+Q^2)}\times\\
&\{g_2(-\mathbf{k},\mathbf{Q})\delta(\mathbf{k}'+\mathbf{k})\,\delta(\mathbf{p}'-\mathbf{Q})+\\
&2\left[g_0\left(\frac{\mathbf{p}+\mathbf{Q}}{\sqrt{2}},-\frac{\mathbf{k}+\mathbf{p}-\mathbf{Q}}{\sqrt{2}}\right)+\frac{1}{6}g_2\left(\frac{\mathbf{p}+\mathbf{Q}}{\sqrt{2}},-\frac{\mathbf{k}+\mathbf{p}-\mathbf{Q}}{\sqrt{2}}\right)\right]\delta\left(\frac{\mathbf{p}+\mathbf{Q}}{\sqrt{2}}-\mathbf{k}'\right)\delta\left(\frac{\mathbf{k}+\mathbf{p}-\mathbf{Q}}{\sqrt{2}}+\mathbf{p}'\right)\\
&+2\left[g_0\left(\frac{\mathbf{p}-\mathbf{Q}}{\sqrt{2}},\frac{\mathbf{k}+\mathbf{p}+\mathbf{Q}}{\sqrt{2}}\right)+\frac{1}{6}g_2\left(\frac{\mathbf{p}-\mathbf{Q}}{\sqrt{2}},\frac{\mathbf{k}+\mathbf{p}+\mathbf{Q}}{\sqrt{2}}\right)\right]\delta\left(\frac{\mathbf{p}-\mathbf{Q}}{\sqrt{2}}-\mathbf{k}'\right)\delta\left(\frac{\mathbf{k}+\mathbf{p}+\mathbf{Q}}{\sqrt{2}}-\mathbf{p}'\right)\bigg\},
\end{aligned}$$

For the numerical integration of Eqs. (70), we represent them in the following form (the index n below represents the number of the interation)

$$g_{0,n+1}(\mathbf{k},\mathbf{p}) = \int(d\mathbf{k}')\left\{P_{00}(\mathbf{k},\mathbf{k}')g_{0,n}(\mathbf{k}')+P_{02}(\mathbf{k},\mathbf{k}')g_{2,n}(\mathbf{k}')\right\}, \tag{70}$$

$$g_{2,n+1}(\mathbf{k},\mathbf{p}) = \int(d\mathbf{k}')\left\{P_{20}(\mathbf{k},\mathbf{k}')g_{0,n}(\mathbf{k}')+P_{22}(\mathbf{k},\mathbf{k}')g_{2,n}(\mathbf{k}')\right\}+\frac{\lambda_2}{\ln[(2+\epsilon)(1+k^2+p^2)]}g_{2,n}(\mathbf{k},\mathbf{p}), \tag{71}$$

where

$$\begin{aligned}
P_{00}(\mathbf{k},\mathbf{p};\mathbf{k}',\mathbf{p}') &= P^I(\mathbf{k},\mathbf{p};\mathbf{k}',\mathbf{p}')+\frac{2}{3}\left(P^{II}(\mathbf{k},\mathbf{p};\mathbf{k}',\mathbf{p}')+P^{III}(\mathbf{k},\mathbf{p};\mathbf{k}',\mathbf{p}')\right),\\
P_{02}(\mathbf{k},\mathbf{p};\mathbf{k}',\mathbf{p}') &= \frac{10}{9}\left(P^{II}(\mathbf{k},\mathbf{p};\mathbf{k}',\mathbf{p}')+P^{III}(\mathbf{k},\mathbf{p};\mathbf{k}',\mathbf{p}')\right),\\
P_{20}(\mathbf{k},\mathbf{k}') &= 2\left(P^{II}(\mathbf{k},\mathbf{p};\mathbf{k}',\mathbf{p}')+P^{III}(\mathbf{k},\mathbf{p};\mathbf{k}',\mathbf{p}')\right),\\
P_{22}(\mathbf{k},\mathbf{p};\mathbf{k}',\mathbf{p}') &= P^I(\mathbf{k},\mathbf{p};\mathbf{k}',\mathbf{p}')+\frac{1}{3}\left(P^{II}(\mathbf{k},\mathbf{p};\mathbf{k}',\mathbf{p}')+P^{III}(\mathbf{k},\mathbf{p};\mathbf{k}',\mathbf{p}')\right),
\end{aligned} \tag{72}$$

and

$$P^{I}(\mathbf{k},\mathbf{p};\mathbf{k}',\mathbf{p}') = \int \frac{d^2\mathbf{Q}}{(2\pi)^2}\frac{4\pi[2+\epsilon+(k')^2+(p')^2]}{\ln[2+\epsilon+(k')^2+(p')^2]}\frac{\delta(\mathbf{p}'-\mathbf{Q})\,\delta(\mathbf{k}'+\mathbf{k})}{(2+\epsilon+k^2+p^2)(1+(k')^2+(p')^2+p^2)}, \tag{73}$$

$$P^{II}(\mathbf{k},\mathbf{p};\mathbf{k}',\mathbf{p}') = \int \frac{d^2\mathbf{Q}}{(2\pi)^2}\frac{4\pi[2+\epsilon+(k')^2+(p')^2]}{\ln[2+\epsilon+(k')^2+(p')^2]}\frac{\delta\left[\mathbf{k}-\left(2\mathbf{Q}-\sqrt{2}(\mathbf{k}'+\mathbf{p}')\right)\right]\delta\left[\mathbf{p}-\left(\sqrt{2}\mathbf{k}'-\mathbf{Q}\right)\right]}{(2+\epsilon+k^2+p^2)(2+\epsilon+k^2+p^2+Q^2)}, \tag{74}$$

$$P^{III}(\mathbf{k},\mathbf{p};\mathbf{k}',\mathbf{p}') = \int \frac{d^2\mathbf{Q}}{(2\pi)^2}\frac{4\pi[2+\epsilon+(k')^2+(p')^2]}{\ln[2+\epsilon+(k')^2+(p')^2]}\frac{\delta\left[\mathbf{k}-\left(\sqrt{2}(\mathbf{p}'+\mathbf{k}')-2\mathbf{Q}\right)\right]\delta\left[\mathbf{p}-\left(\sqrt{2}\mathbf{k}'+\mathbf{Q}\right)\right]}{(2+\epsilon+k^2+p^2)(2+\epsilon+k^2+p^2+Q^2)}. \tag{75}$$

The symmetry of Eqs. (74) and (75) suggests, that all the results for $P^{III}(\mathbf{k},\mathbf{p};\mathbf{k}',\mathbf{p}')$ can be obtained from the results for $P^{II}(\mathbf{k},\mathbf{p};\mathbf{k}',\mathbf{p}')$ by changing $\mathbf{p}'\to-\mathbf{p}'$, $\mathbf{k}'\to-\mathbf{k}'$: $P^{III}(\mathbf{k},\mathbf{p};\mathbf{k}',\mathbf{p}') = P^{II}(\mathbf{k},\mathbf{p};-\mathbf{k}',-\mathbf{p}')$.

4.1 Probability distribution for the kernel P^I

Integration over the vector $\mathbf{Q}$ in Eq. (73) results in the simplified kernel

$$P^{I}(\mathbf{k},\mathbf{p};\mathbf{k}',\mathbf{p}') = \frac{4\pi[2+\epsilon+(k')^2+(p')^2]}{\ln[2+\epsilon+(k')^2+(p')^2]}\frac{\delta(\mathbf{k}'+\mathbf{k})}{(2+\epsilon+k^2+p^2)(1+(k')^2+(p')^2+p^2)}, \tag{76}$$

The kernel $P^I(\mathbf{k},\mathbf{p};\mathbf{k}',\mathbf{p}')$ does contain the angular dependence. The correspondent distribution, as given by Eq. (76), just has to be properly normalized to insure the numerical implementation. The normalization factor is given by

$$\Gamma_1(\mathbf{k}',\mathbf{p}') = \int\frac{d^2\mathbf{k}}{(2\pi)^2}\int\frac{d^2\mathbf{p}}{(2\pi)^2}P^I(\mathbf{k},\mathbf{p};\mathbf{k}',\mathbf{p}') = \frac{2+\epsilon+\mathbf{k}'^2+\mathbf{p}'^2}{1+\epsilon-\mathbf{p}'^2}\left[\frac{\ln(2+\epsilon+\mathbf{k}'^2)-\ln(1+\mathbf{k}'^2+\mathbf{p}'^2)}{\ln(2+\epsilon+\mathbf{k}'^2+\mathbf{p}'^2)}\right]. \tag{77}$$

The corresponding distribution can be then represented as

$$P^I(\mathbf{k},\mathbf{p};\mathbf{k}',\mathbf{p}') = \Gamma_1(\mathbf{k}',\mathbf{p}')W^I(\mathbf{p}^2,\mathbf{k}',\mathbf{p}'), \tag{78}$$

where the probability $W^I(\mathbf{p}^2,\mathbf{k}',\mathbf{p}')$ is given by

$$W^I(\mathbf{p}^2,\mathbf{k}',\mathbf{p}') = \frac{\mathbf{p}'^2}{\ln(2+\epsilon+\mathbf{k}'^2+\mathbf{p}'^2)-\ln(2+\epsilon+\mathbf{k}'^2)}\frac{1}{(2+\epsilon+\mathbf{k}'^2+\mathbf{p}^2)(2+\epsilon+\mathbf{k}'^2+\mathbf{p}'^2+\mathbf{p}^2)}. \tag{79}$$

4.2 Probability distributions for the kernels P^{II} and P^{III}

To facilitate the numerical implementation of integral kernels, it is convenient of get rid of the angular integration by using the Feynman representation of the terms containing the vector $\mathbf{Q}$.

$$\frac{1}{(2+\epsilon+k^2+p^2)(2+\epsilon+k^2+p^2+Q^2)} = \int_0^1\frac{ds}{\left[s(2+\epsilon+k^2+p^2)+(1-s)(2+\epsilon+k^2+p^2+Q^2)\right]^2}. \tag{80}$$

Applying the δ-functions in the integration kernel P^{II}, Eq. (74), and regrouping the terms, we obtain

$$\int\frac{d^2\mathbf{k}}{(2\pi)^2}\int\frac{d^2\mathbf{p}}{(2\pi)^2}\frac{\delta\left[\mathbf{k}-\left(2\mathbf{Q}-\sqrt{2}(\mathbf{k}'+\mathbf{p}')\right)\right]\delta\left[\mathbf{p}-\left(\sqrt{2}\mathbf{k}'-\mathbf{Q}\right)\right]}{(2+\epsilon+k^2+p^2)(2+\epsilon+k^2+p^2+Q^2)} = \int_0^1\frac{ds}{\left[\beta(s)[\mathbf{Q}-\mathbf{P}(s)]^2+\alpha(s)\right]^2}, \tag{81}$$

where

$$\beta(s) = 6-s, \tag{82}$$

$$\alpha(s) = 2+\epsilon+2\left[(\mathbf{k}'+\mathbf{p}')^2+k'^2\right]-\frac{2}{6-s}(3\mathbf{k}'+2\mathbf{p}')^2, \tag{83}$$

$$\mathbf{P}(s) = \frac{\sqrt{2}}{6-s}(3\mathbf{k}'+2\mathbf{p}'). \tag{84}$$

Using the Feynman integral (80), we represent the kernel P^{II} in the form

$$P^{II}(\mathbf{k},\mathbf{p};\mathbf{Q},\mathbf{k}',\mathbf{p}') = \frac{4\pi[2+\epsilon+k'^2+p'^2]}{\ln[2+\epsilon+k'^2+p'^2]}\int_0^1 \frac{ds}{[\beta(s)[\mathbf{Q}-\mathbf{P}(s)]^2+\alpha(s)]^2}\times$$
$$\delta\left[\mathbf{k}-\left(2\mathbf{Q}-\sqrt{2}(\mathbf{k}'+\mathbf{p}')\right)\right]\delta\left[\mathbf{p}-\left(\sqrt{2}\mathbf{k}'-\mathbf{Q}\right)\right]. \tag{85}$$

4.3 The probability distributions $w(s)$ and $P_s(r^2,\mathbf{k}',\mathbf{p}')$

The kernel $P^{II}(\mathbf{k},\mathbf{p};\mathbf{Q},\mathbf{k}',\mathbf{p}')$ in Eq. (85) is not normalized, and therefore it cannot be directly interpreted as a probability distribution. The normalization factor can be calculated as

$$\Gamma_2(\mathbf{k}',\mathbf{p}') = \int \frac{d^2\mathbf{k}}{(2\pi)^2}\frac{d^2\mathbf{p}}{(2\pi)^2}\frac{d^2\mathbf{Q}}{(2\pi)^2}P^{II}(\mathbf{k},\mathbf{p};\mathbf{Q},\mathbf{k}',\mathbf{p}') =$$
$$\frac{4\pi[2+\epsilon+k'^2+p'^2]}{\ln[2+\epsilon+k'^2+p'^2]}\int_0^1 ds\int\frac{d^2\mathbf{Q}}{(2\pi)^2}\frac{1}{[\beta(s)[\mathbf{Q}-\mathbf{P}(s)]^2+\alpha(s)]^2} \tag{86}$$

Performing the integration over $\mathbf{Q}$ results in

$$\int\frac{d^2\mathbf{Q}}{(2\pi)^2}\frac{1}{[\beta(s)[\mathbf{Q}-\mathbf{P}(s)]^2+\alpha(s)]^2} = \frac{1}{4\pi\alpha(s)\beta(s)} \tag{87}$$

Further integration over s gives

$$\int_0^1 \frac{ds}{4\pi\alpha(s)\beta(s)} = \frac{1}{4\pi A(\mathbf{k}',\mathbf{p}')}\ln\left|\frac{6A(\mathbf{k}',\mathbf{p}')-B(\mathbf{k}',\mathbf{p}')}{6A(\mathbf{k}',\mathbf{p}')-B(\mathbf{k}',\mathbf{p}')-1}\right|, \tag{88}$$

where

$$A(\mathbf{k}',\mathbf{p}') = 2+\epsilon+2\left[(\mathbf{k}'+\mathbf{p}')^2+k'^2\right], \tag{89}$$
$$B(\mathbf{k}',\mathbf{p}') = 2(3\mathbf{k}'+2\mathbf{p}')^2. \tag{90}$$

Therefore, the total normalization factor is given by

$$\Gamma_2(\mathbf{k}',\mathbf{p}') = \frac{[2+\epsilon+k'^2+p'^2]}{A(\mathbf{k}',\mathbf{p}')\ln[2+\epsilon+k'^2+p'^2]}\ln\left|\frac{6A(\mathbf{k}',\mathbf{p}')-B(\mathbf{k}',\mathbf{p}')}{6A(\mathbf{k}',\mathbf{p}')-B(\mathbf{k}',\mathbf{p}')-1}\right| \tag{91}$$

In order $w(s,\mathbf{k}',\mathbf{p}')$ and $P_s(r^2,\mathbf{k}',\mathbf{p}')$ be the proper distribution functions, the following normalization conditions have to be fulfilled

$$\int_0^\infty \frac{d(r^2)}{4\pi}P_s(r^2,\mathbf{k}',\mathbf{p}') = 1, \tag{92}$$
$$\int_0^s ds\, w(s,\mathbf{k}',\mathbf{p}') = 1. \tag{93}$$

The factor $1/(4\pi)$ in Eq. (92) takes into account the change of the integration measure and angular integration

$$\int\frac{d^2\mathbf{Q}}{(2\pi)^2} = \frac{1}{2\pi}\int_0^\infty QdQ = \frac{1}{4\pi}\int_0^\infty d(r^2). \tag{94}$$

If the two conditions Eqs. (92), (93) are satisfied, the combined distribution $w(s,\mathbf{k}',\mathbf{p}')P_s(r^2,\mathbf{k}',\mathbf{p}')$ is normalized to 1 for each initial point $\mathbf{k}',\mathbf{p}'$. Taking into account Eqs. (87), (88), we obtain for $P_s(r^2,\mathbf{k}',\mathbf{p}')$ and $w(s),\mathbf{k}',\mathbf{p}'$ the following explicit expressions

$$P_s(r^2,\mathbf{k}',\mathbf{p}') = \frac{4\pi\alpha(s)\beta(s)}{[\beta(s)r^2+\alpha(s)]^2},\ (r^2 = [\mathbf{Q}-\mathbf{P}(s)]^2) \tag{95}$$

$$w(s,\mathbf{k}',\mathbf{p}') = \frac{A(\mathbf{k}',\mathbf{p}')}{\ln\left|\frac{6A(\mathbf{k}',\mathbf{p}')-B(\mathbf{k}',\mathbf{p}')}{6A(\mathbf{k}',\mathbf{p}')-B(\mathbf{k}',\mathbf{p}')-1}\right|}\frac{1}{\alpha(s)\beta(s)}. \tag{96}$$

The kernel P^{II} is then given by

$$P^{II}(\mathbf{k},\mathbf{p};\mathbf{Q},\mathbf{k}',\mathbf{p}') = \Gamma_2(\mathbf{k}',\mathbf{p}')\int_0^1 dsw(s,\mathbf{k}',\mathbf{p}')P_s(r^2,\mathbf{k}',\mathbf{p}')\delta\left[\mathbf{k}-\left(2\mathbf{Q}-\sqrt{2}(\mathbf{k}'+\mathbf{p}')\right)\right]\delta\left[\mathbf{p}-\left(\sqrt{2}\mathbf{k}'-\mathbf{Q}\right)\right] \tag{97}$$

with $r^2 = [\mathbf{Q}-\mathbf{P}(s)]^2$. Here $w(s,\mathbf{k}',\mathbf{p}')$ and $P_s(r^2,\mathbf{k}',\mathbf{p}')$ are normalized probability distributions that can be generated numerically.

The distribution P^{III} can be represented in the similar way. The only difference consists in definitions of r^2, and the constants A and B. Namely, one can write

$$P^{III}(\mathbf{k},\mathbf{p};\mathbf{Q},\mathbf{k}',\mathbf{p}') = \Gamma_3(\mathbf{k}',\mathbf{p}')\int_0^1 dsw_3(s,\mathbf{k}',\mathbf{p}')P_{s3}(r^2,\mathbf{k}',\mathbf{p}')\delta\left[\mathbf{k}-\left(\sqrt{2}(\mathbf{p}'+\mathbf{k}')-2\mathbf{Q}\right)\right]\delta\left[\mathbf{p}-\left(\sqrt{2}\mathbf{k}'+\mathbf{Q}\right)\right], \tag{98}$$

where the following definitions have been made

$$P_{s3}(r^2,\mathbf{k}',\mathbf{p}') = \frac{4\pi\alpha_3(s)\beta_3(s)}{[\beta(s)r^2+\alpha(s)]^2},\ r^2 = [\mathbf{Q}-\mathbf{P}_3]^2,\ \mathbf{P}_3 = \frac{\sqrt{2}}{6-s}(3\mathbf{k}'-2\mathbf{p}'), \tag{99}$$

$$w_3(s) = \frac{A_3}{\ln\left|\frac{6A_3(\mathbf{k}',\mathbf{p}')-B_3(\mathbf{k}',\mathbf{p}')}{6A_3(\mathbf{k}',\mathbf{p}')-B_3(\mathbf{k}',\mathbf{p}')-1}\right|}\frac{1}{\alpha_3(s)\beta_3(s)}, \tag{100}$$

$$\beta_3(s) = 6-s,\ \alpha_3(s) = 2+\epsilon+2\left[(\mathbf{k}'-\mathbf{p}')^2+k'^2\right]-\frac{2}{6-s}(3\mathbf{k}'-2\mathbf{p}')^2, \tag{101}$$

$$A_3 = 2+\epsilon+2\left[(\mathbf{k}'-\mathbf{p}')^2+k'^2\right],\ B_3 = 2(3\mathbf{k}'-2\mathbf{p}')^2, \tag{102}$$

$$\Gamma_3(\mathbf{k}',\mathbf{p}') = \frac{[2+\epsilon+k'^2+p'^2]}{A(\mathbf{k}',\mathbf{p}')\ln[2+\epsilon+k'^2+p'^2]}\ln\left|\frac{6A_3(\mathbf{k}',\mathbf{p}')-B_3(\mathbf{k}',\mathbf{p}')}{6A_3(\mathbf{k}',\mathbf{p}')-B_3(\mathbf{k}',\mathbf{p}')-1}\right|. \tag{103}$$

4.4 Master equation for 2-channel model

To construct the master equation for the 2-channel model described by Eqs. (70), (71), we consider the following process: consider two flavors of particles, dubbed by "0" and "2" (pseudospin language can be used as well), moving in the four-dimensional $\mathbf{k}$-space, here $\mathbf{k} = (\mathbf{k}_x,\mathbf{k}_z)$. The following elementary processes can occur at each discrete time step: (i) a particle of the sort ν jumps from the position $\mathbf{k}'$ to the position $\mathbf{k}$ without changing its flavor with probability $W_{\nu\nu'}(\mathbf{k},\mathbf{k}')$, thereby it keeps its flavor if $\nu'=\nu$ and changes the flavor from ν' to ν, if the two are different. (ii) A particle can be annihilated with the probability $\gamma_\nu(\mathbf{k}')$ (if $\gamma_\nu(\mathbf{k}')<0$, another particle is created at the position $\mathbf{k}'$ with the probability $|\gamma_\nu(\mathbf{k}')|$. The corresponding master equations read

$$g_{\nu,n+1}(\mathbf{k}) - g_{\nu,n}(\mathbf{k}) = \sum_{\nu'}\int(d\mathbf{k}')\left\{W_{\nu\nu'}(\mathbf{k},\mathbf{k}')g_{\nu',n}(\mathbf{k}') - W_{\nu'\nu}(\mathbf{k}',\mathbf{k})g_{\nu,n}(\mathbf{k})\right\} - \gamma_\nu(\mathbf{k})g_{\nu,n}(\mathbf{k}). \tag{104}$$

To make a relation between Eqs. (70), (71), and Eq. (104), we divide RHS of the former equations by a constant C, which is to be chosen in such a way, that the kernels in RHS have the meaning of probabilities. This requirement results in the following conditions for the constant C

$$C = \max_{\{\mathbf{k}\}}\left\{\Gamma_{02}(\mathbf{k})+\Gamma_{22}(\mathbf{k}),\Gamma_{00}(\mathbf{k})+\Gamma_{20}(\mathbf{k})\right\} = \max\left(\bar{\Gamma}_1+\frac{26}{9}\bar{\Gamma}_2+\frac{\lambda_2}{\ln(2+\epsilon)},\bar{\Gamma}_1+\frac{16}{3}\bar{\Gamma}_2\right). \tag{105}$$

where we denoted

$$\Gamma_{ij}(\mathbf{k}) = \int d\mathbf{k}'P_{ij}(\mathbf{k}',\mathbf{k}). \tag{106}$$

Let us also denote

$$\kappa_2(\mathbf{k}) = \frac{\lambda_2}{\ln(2+\epsilon+k^2)}. \tag{107}$$

4.5 Upper bounds for Γ, determination of C

To determine the appropriate constant C, we look for the majorants of Γ_1, Γ_2, and Γ_3 as functions of $\mathbf{k}'$.

4.5.1 Upper bound for Γ_2

We start with Γ_2, as given by Eq.(91). We use the parametrization of the components through the absolute value of the vector k', and the angle φ between the $\mathbf{k'}_x$ and $\mathbf{k'}_z$:

$$k'_x = k'\cos\varphi,\;\; k'_z = k'\sin\varphi. \tag{108}$$

Then the function $A(\mathbf{k}')$ given by Eq. (89) can be represented as

$$A(\mathbf{k}') = 1 + 2(k')^2\left[1+\cos^2\varphi + \sin(2\varphi)\right] \tag{109}$$

Estimating the supremum and infimum of Eq. (109) with respect to φ, we obtain

$$1 + 0.763(k')^2 \le A(\mathbf{k}') \le 1 + 3.237(k')^2 \tag{110}$$

Furthermore, the combination $6A - B$, entering the logarithm, can be represented as

$$6A - B = 6 + 2(k')^2\left(2+\cos^2\varphi\right) \tag{111}$$

Evaluating the maximum and the minimum with respect to φ, we obtain the estimation

$$6 + 4(k')^2 \le 6A - B \le 6 + 6(k')^2. \tag{112}$$

For $5A - B$ we obtain

$$5A - B = 5 + 2(k')^2\left(1 - \sin 2\varphi\right), \tag{113}$$

and

$$5 \le 5A - B \le 5 + 4(k')^2. \tag{114}$$

Using the estimations above, we arrive at the following upper boundary for Γ_2 as a function of $(k')^2$

$$\Gamma_2(k') \le \frac{1+(k')^2}{(1+0.763(k')^2)\ln(2+\epsilon+(k')^2)}\ln\left[\frac{6+6(k')^2}{5}\right]. \tag{115}$$

Investigation of Eq. (115) as a function of $(k')^2$ allows estimation of its upper bound as

$$\Gamma_2(k') < 1.36 \tag{116}$$

Since Γ_3 and Γ_2 are related by transformation $\mathbf{k'}_z \to -\mathbf{k'}_z$, the bound Eq. (116) is also an upper bound for Γ_3.

4.5.2 Upper bound for Γ_1

Using the "radius-angle" parametrization Eq. (108), the rate Γ_1, Eq. (77) can be written as

$$\Gamma_1 = \frac{1+(k')^2}{(k')^2 \sin^2\varphi \ln[(2+\epsilon)(1+(k')^2)]} \ln\left[\frac{1+(k')^2}{1+(k')^2\cos^2\varphi}\right]. \tag{117}$$

Analysis of the angular dependence shown, that Eq. (117) reaches its maximal value at $\sin^2\varphi = 1$. At that point we have

$$\bar{\Gamma}_1(k') = \frac{1+(k')^2}{(k')^2 \ln[(2+\epsilon)(1+(k')^2)]} \ln\left(1+(k')^2\right). \tag{118}$$

The maximum of this expression is reached either at $k' = 0$, in which case it equals $1/\ln(2+\epsilon)$, or in the limit $k' \to \infty$, in which case it is given by 1. Therefore, the upper bound for Γ_1 can be determined as

$$\Gamma_1 \leq \max\left\{1, \frac{1}{\ln(2+\epsilon)}\right\}. \tag{119}$$

Taking into account the relations Eqs. (72) and Eqs. (116), (119), we obtain

$$C = \max\left\{\max\left(1, \frac{1}{\ln(2+\epsilon)}\right) + 3.93 + \frac{\lambda_2}{\ln(2+\epsilon)}, \max\left(1, \frac{1}{\ln(2+\epsilon)}\right) + 7.25\right\} \tag{120}$$

4.6 Numerical algorithm

Given the point $\mathbf{k}'$ and the particle of the sort ν, generate the number $i = 1, 2, 3$ with the probability $\Gamma_{\nu\nu}(\mathbf{k}')/C$, $\Gamma_{\bar{\nu}\nu}(\mathbf{k}')/C$ and $|1 - \Gamma_{\nu\nu}(\mathbf{k}') - \Gamma_{\bar{\nu}\nu}(\mathbf{k}') - \kappa_2(\mathbf{k}')\delta_{\nu 2}|/C$ respectively, otherwise generate the number 4. Further steps are performed using the algorithm described for the single-channel model.
If $\nu = 0$:
for $i = 1$ perform a jump to the position $\mathbf{k}$ with probability $P_{00}(\mathbf{k}, \mathbf{k}')/C$;
for $i = 2$ perform a jump to the position $\mathbf{k}$ with probability $P_{20}(\mathbf{k}, \mathbf{k}')/C$;
for $i = 3$, destroy the particle (create the particle if $\gamma_0(\mathbf{k}') < 0$);
for $i = 4$ do nothing.
If $\nu = 2$:
for $i = 1$ perform a jump to the position $\mathbf{k}$ with probability $P_{22}(\mathbf{k}, \mathbf{k}')/C$;
for $i = 2$ perform a jump to the position $\mathbf{k}$ with probability $P_{02}(\mathbf{k}, \mathbf{k}')/C$;
for $i = 3$: For $\gamma_2(\mathbf{k}') > 0$, destroy a particles of the sort 2 at the position $\mathbf{k}'$. For $\gamma_2(\mathbf{k}') < 0$, create a particle;
for $i = 4$ do nothing.

Mathematica notebook script implementing that algorithm is provided in Ref. [4].

References

[1] D. S. Petrov, and G. V. Shlyapnikov, Phys. Rev. A **64**, 012706 (2001).

[2] Z. Idziaszek, K. Jachymski, P. S. Julienne, New Journal of Physics, **17**, 1 (2015).

[3] D. S. Petrov, "The few atom problem", arXiv:1206.5752v2 (2012).

[4] https://drive.google.com/open?id=11wdTyWEzRgTX8B2DEqJe9jURr7z9o0JV
